# Supplementary material for: Bacteriophages Isolated from Stunted Children Can Regulate Gut Bacterial Communities in an Age-Specific Manner
Source: Cell Host Microbe. 2020 Feb 12;27(2):199–212.e5. doi: 10.1016/j.chom.2020.01.004 (PMC7013830; doi:10.1016/j.chom.2020.01.004)
Supplement: Document S1. Figures S1–S8 [file mmc1.pdf]

Cell Host & Microbe, Volume *27*

Supplemental Information

Bacteriophages Isolated from Stunted Children

Can Regulate Gut Bacterial Communities

in an Age-Specific Manner

Mohammadali Khan Mirzaei, Md. Anik Ashfaq Khan, Prakash Ghosh, Zofia E. Taranu, Mariia Taguer, Jinlong Ru, Rajashree Chowdhury, Md. Mamun Kabir, Li Deng, Dinesh Mondal, and Corinne F. Maurice

SUPPLEMENTAL FIGURES

Figure S1. Related to all Figures. (a) Overview of experimental procedures. Schematic diagram of the experimental setup and procedures for one age group. These procedures were repeated in a second age group, leading to a total of 60 children surveyed. (b) Metagenomics workflow, with color-coded different types of analytical steps. * A cut-off value of e-value <1e-5 was used. When contigs showed similarity to different strains within one species, a common ancestor was used. ⌘ indicates that >70% coverage and >90% identity were used.

Figure S2. Related to Figures 1 and 3. Viral and bacterial abundances are similar across health status and age. Violin plots of whole community abundances of (a) Virus-Like Particles (VLPs), (b) Bacteria, and (c) the resulting Virus-to-bacteria ratio in fecal samples from 30 non-stunted and 30 stunted children aged 14-38 months. The enumeration of phage and bacterial cells was performed using epifluorescence microscopy (see Star Methods). N= 60 children total (15/age group/health status). The width of the violin plots reflects the frequency distribution of the data, dotted lines show the median and lower and upper quartiles.

Figure S3. Related to Figure 1. Shannon diversity (a, b) and richness (c, d) indices for bacterial phyla in the original samples show opposing patterns from those observed at the species level. Each index is compared among (a, c) the age according to the children's health status, and (b, d) the HAZ score according to the children's age group. In all cases, there is an interaction between the children's age and health status on Shannon diversity and richness indexes. In particular, we see that richness increases with HAZ, whereas Shannon decreases. Pools I-III: samples from the same age group were pooled randomly for sequencing (pool size was 3-5 samples).

Figure S4. Related to Figure 5. Changes in bacterial phyla Shannon evenness and richness in cross-infected cohorts among age groups. Shannon diversity (a) and richness (b) indices for bacterial phyla. Bacterial evenness decreased with age in stunted children (a), whereas the number of phyla either increased (stunted, green tones) or remained stable (non-stunted, red tones) with age (b).

Figure S5. Related to Figure 5. **Local contribution of samples to β-phage species diversity (LCBD) after cross-infections**. Shown are the LCBD *vs.* treatment type for the younger (top) and the older (bottom) age groups, where cross-infection samples with non-stunted bacteria are shown in red and those with stunted bacteria are in green.

Figure S6. Related to Figure 6. Multiple Factor Analysis of all bacterial species, phage species, and children's metadata in younger children. Quantitative variables: milk feed, age, sex, HAZ, WAZ, and WHZ; qualitative variables: health status, treatment type, and treatment factor. Shown in the first panel is the ordination of qualitative and in the second and third panel the contribution of the quantitative variables to dimensions 1 and 2, respectively, where the dashed red line separates the variables that contribute the most (higher than average contribution of all variables) to the MFA. The Hellinger transformation of the bacteria species, phage species, and phage replication cycle data are presented here.

Figure S7. Related to Figure 6. Multiple Factor Analysis of all bacterial species, phage species, and children's metadata in older children. Quantitative variables: milk feed, age, sex, HAZ, WAZ, and WHZ; qualitative variables: health status, treatment type, and treatment factor. Shown in the first panel is the ordination of qualitative and in the second and third panel the contribution of the quantitative variables to dimensions 1 and 2, respectively, where the dashed red line separates the variables that contribute the most (higher than average contribution of all variables) to the MFA. The Hellinger transformation of the bacteria species, phage species, and phage replication cycle data are presented here.

Figure S8. Related to Figure 7. Phage communities alter functional traits of bacterial community in an age-specific manner. (a) MFA of the dominant functional traits and children metadata for both age groups. Dominant functional traits shown were selected using a PCA and the contribution circle from the cleanplot.pca() function. MFA ordination of the qualitative variables (health status, treatment type, and treatment factor). The Hellinger transformation of the bacterial, phage, and replication cycle data are presented here. (b) Relationship between the local contribution of samples to beta diversity of the functional traits (LCBD) after cross-infections for bacteria from (top panels) and phages (bottom panels).

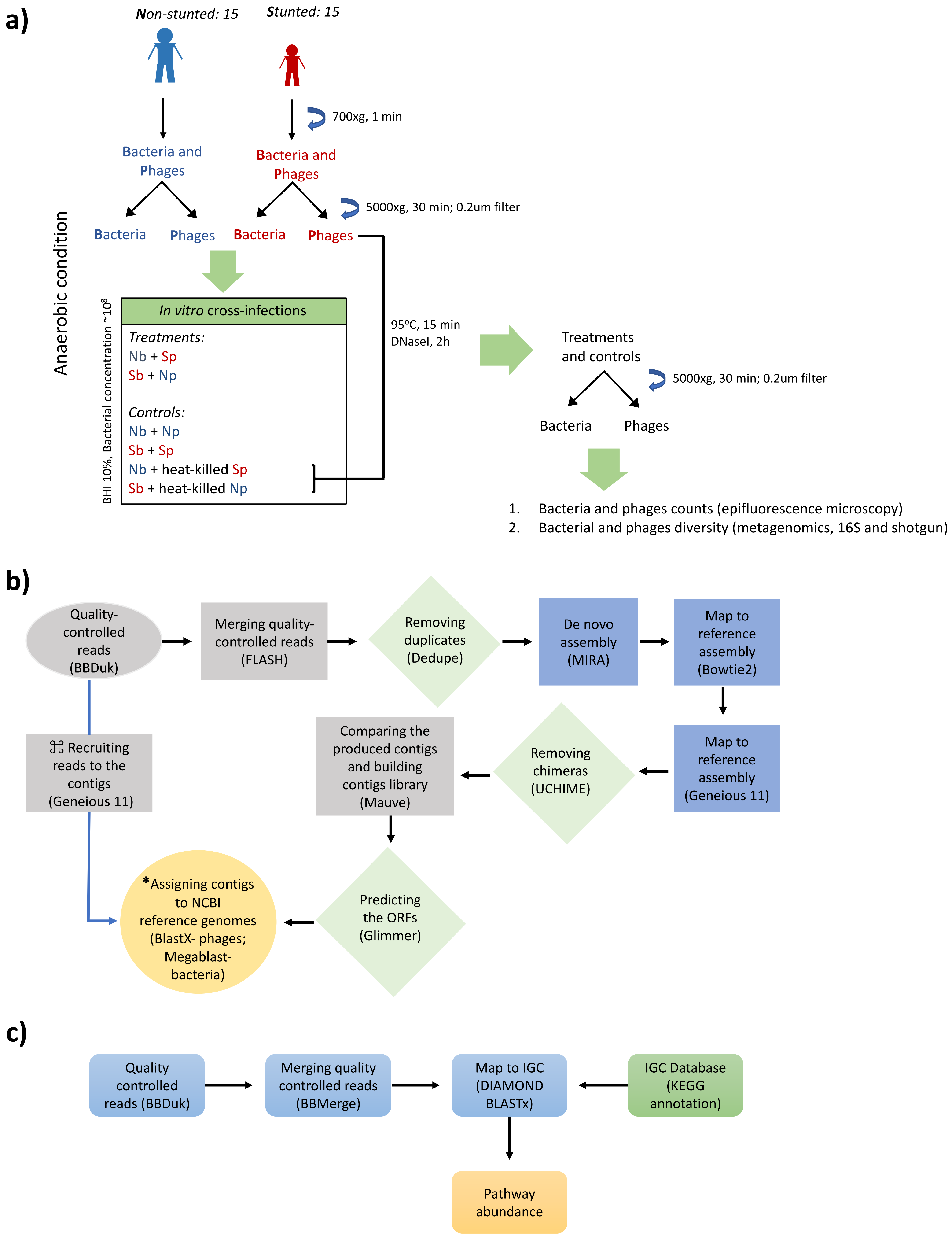

a)
Non-stunted: 15
Stunted: 15
700xg, 1 min
Bacteria and Phages
Bacteria and Phages
5000xg, 30 min; 0.2um filter
Bacteria
Phages
Bacteria
Phages
Anaerobic condition
BHI 10%, Bacterial concentration ~10^8
In vitro cross-infections
Treatments:
Nb + Sp
Sb + Np
Controls:
Nb + Np
Sb + Sp
Nb + heat-killed Sp
Sb + heat-killed Np
95ºC, 15 min
DNaseI, 2h
Treatments and controls
5000xg, 30 min; 0.2um filter
Bacteria
Phages
1. Bacteria and phages counts (epifluorescence microscopy)
2. Bacterial and phages diversity (metagenomics, 16S and shotgun)
b)
Quality-controlled reads (BBDuk)
Merging quality-controlled reads (FLASH)
Removing duplicates (Dedupe)
De novo assembly (MIRA)
Map to reference assembly (Bowtie2)
⌘ Recruiting reads to the contigs (Geneious 11)
Comparing the produced contigs and building contigs library (Mauve)
Removing chimeras (UCHIME)
Map to reference assembly (Geneious 11)
*Assigning contigs to NCBI reference genomes (BlastX- phages; Megablast-bacteria)
Predicting the ORFs (Glimmer)
c)
Quality controlled reads (BBDuk)
Merging quality controlled reads (BBMerge)
Map to IGC (DIAMOND BLASTx)
IGC Database (KEGG annotation)
Pathway abundance

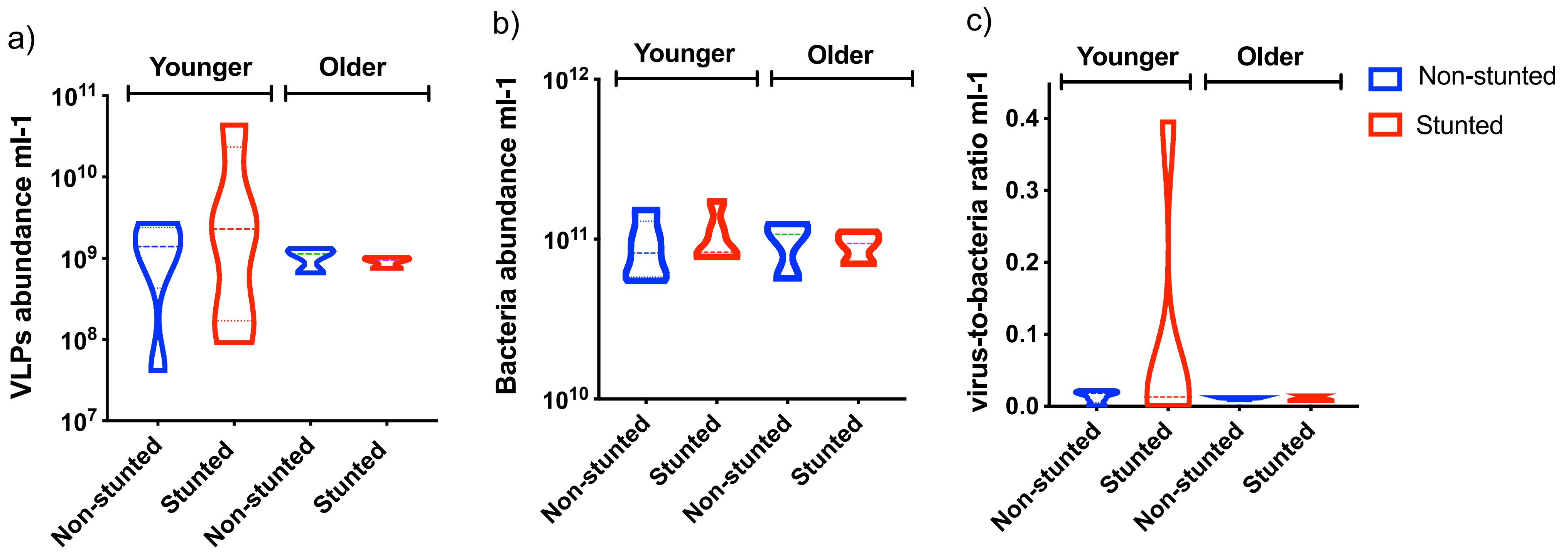
a)
Younger
Older
VLPs abundance ml-1
10^{11}
10^{10}
10^{9}
10^{8}
10^{7}
Non-stunted
Stunted
Non-stunted
Stunted
b)
Younger
Older
Bacteria abundance ml-1
10^{12}
10^{11}
10^{10}
Non-stunted
Stunted
Non-stunted
Stunted
c)
Younger
Older
virus-to-bacteria ratio ml-1
0.4
0.3
0.2
0.1
0.0
Non-stunted
Stunted
Non-stunted
Stunted
Non-stunted
Stunted

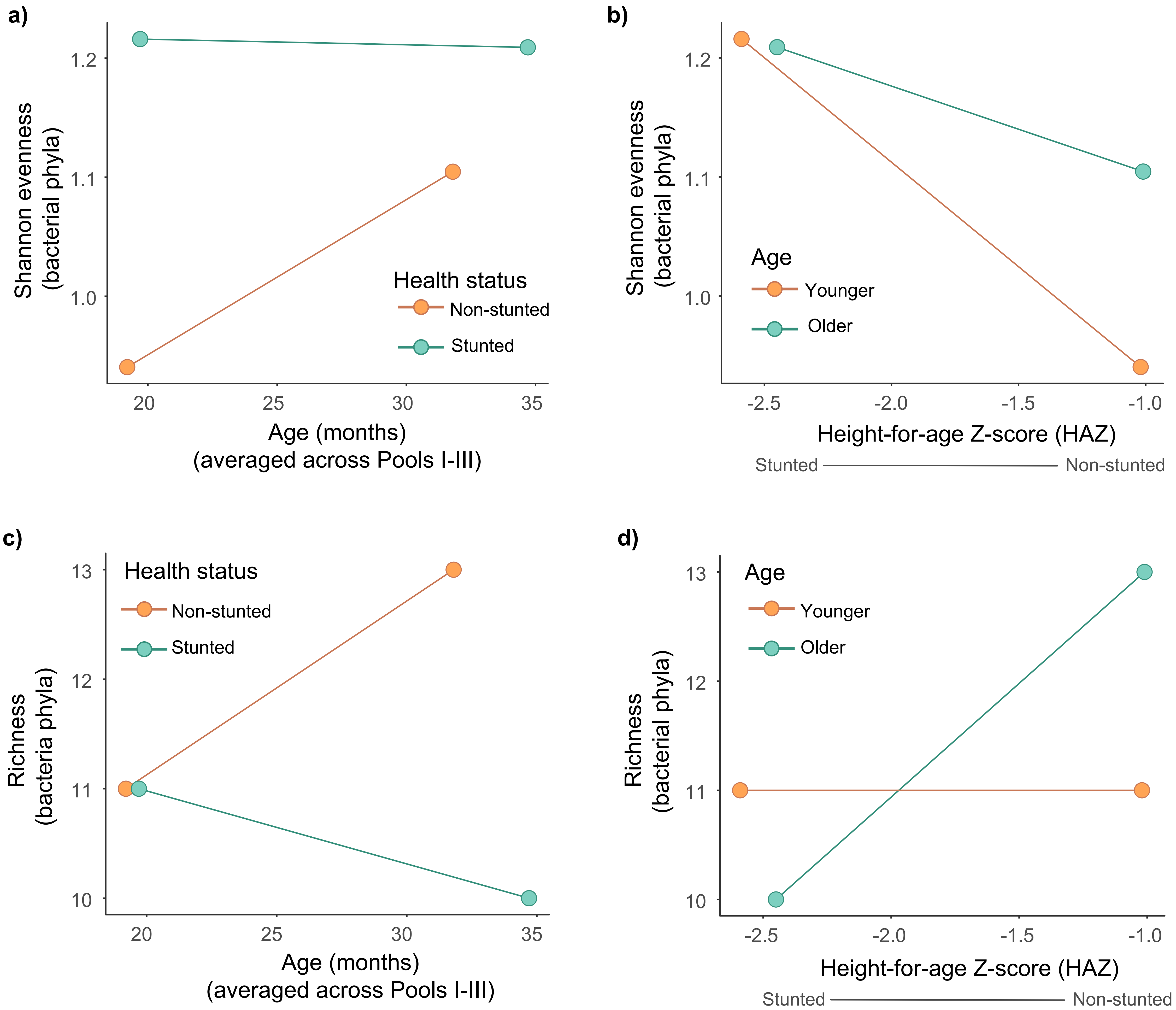

a)
Shannon evenness
(bacterial phyla)
1.2
1.1
1.0
Health status
Non-stunted
Stunted
20
25
30
35
Age (months)
(averaged across Pools I-III)
b)
Shannon evenness
(bacterial phyla)
1.2
1.1
1.0
Age
Younger
Older
-2.5
-2.0
-1.5
-1.0
Height-for-age Z-score (HAZ)
Stunted
Non-stunted
c)
Richness
(bacteria phyla)
13
12
11
10
Health status
Non-stunted
Stunted
20
25
30
35
Age (months)
(averaged across Pools I-III)
d)
Richness
(bacterial phyla)
13
12
11
10
Age
Younger
Older
-2.5
-2.0
-1.5
-1.0
Height-for-age Z-score (HAZ)
Stunted
Non-stunted

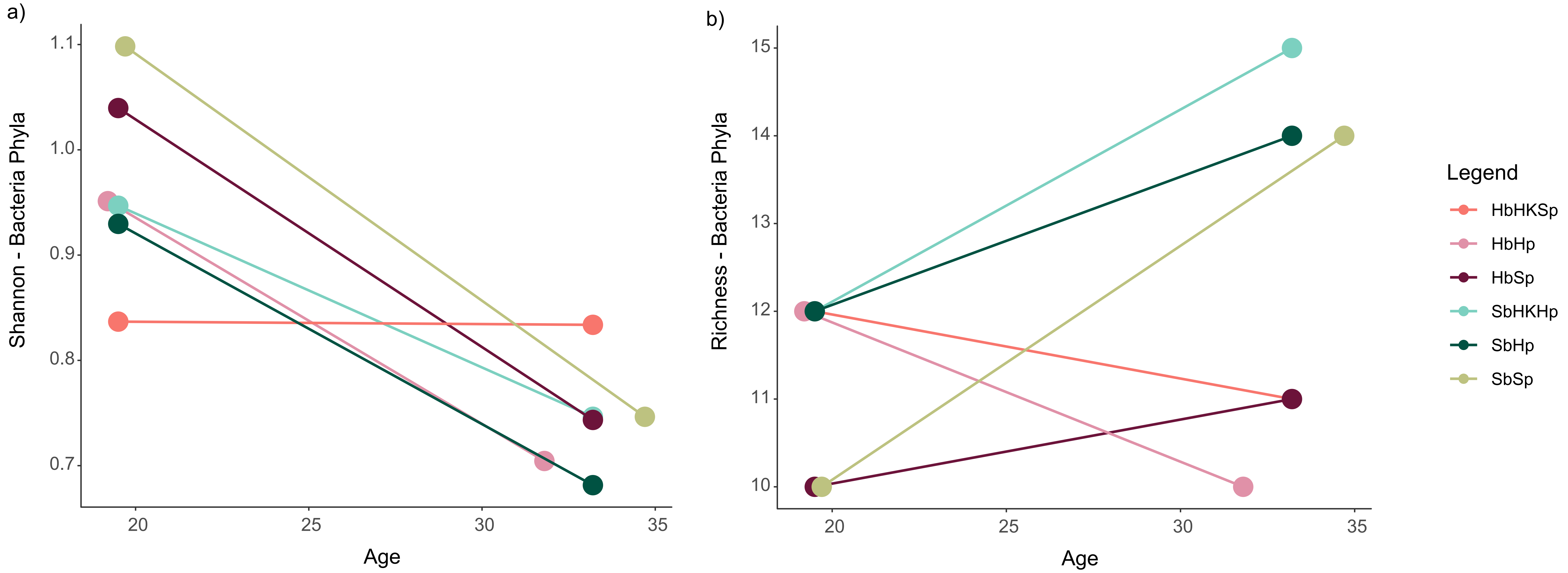
a)
Shannon - Bacteria Phyla
1.1
1.0
0.9
0.8
0.7
20
25
30
35
Age
b)
Richness - Bacteria Phyla
15
14
13
12
11
10
20
25
30
35
Age
Legend
HbHKSp
HbHp
HbSp
SbHKHp
SbHp
SbSp

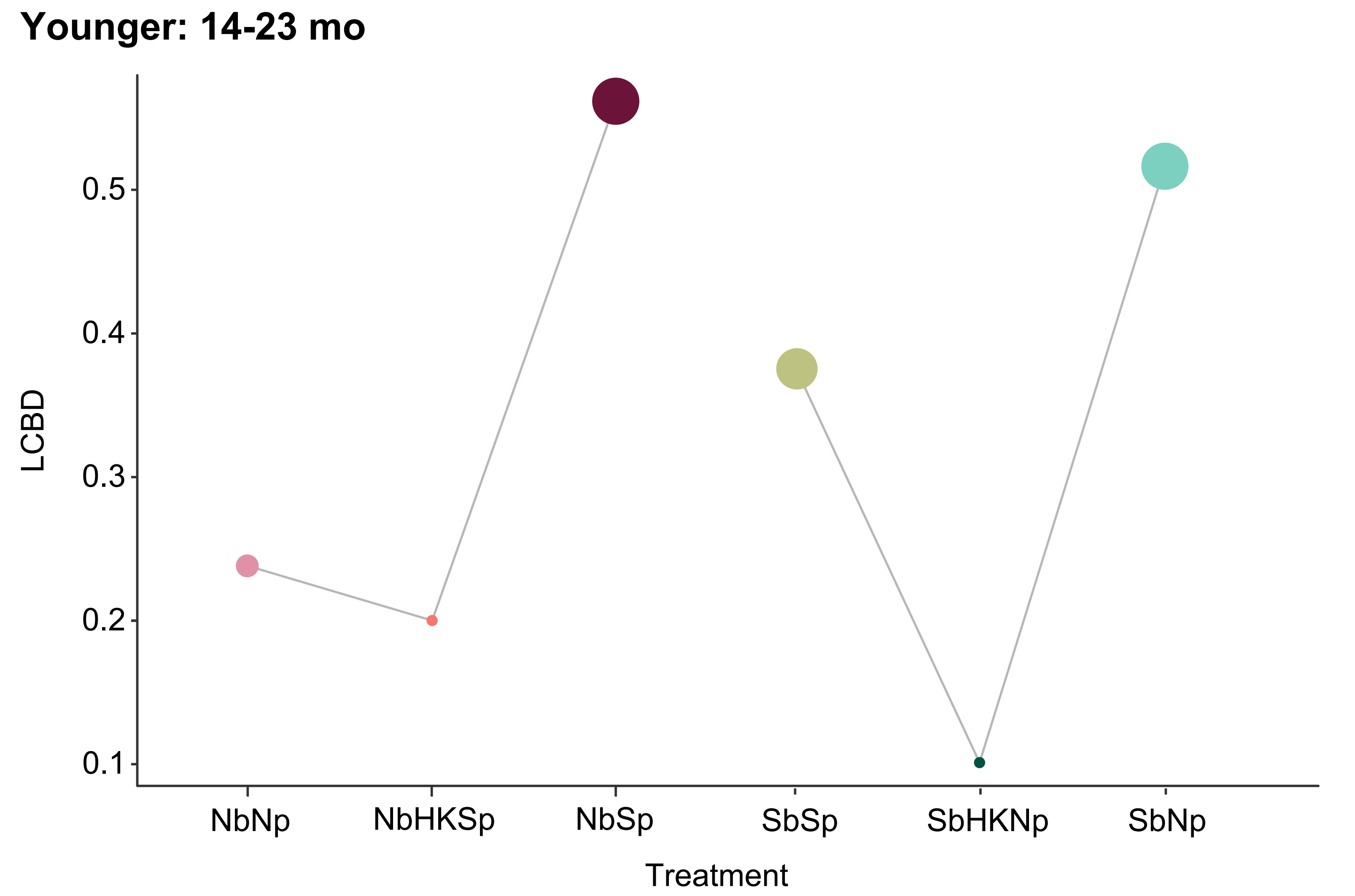
Younger: 14-23 mo
LCBD
0.1
0.2
0.3
0.4
0.5
NbNp
NbHKSp
NbSp
SbSp
SbHKNp
SbNp
Treatment

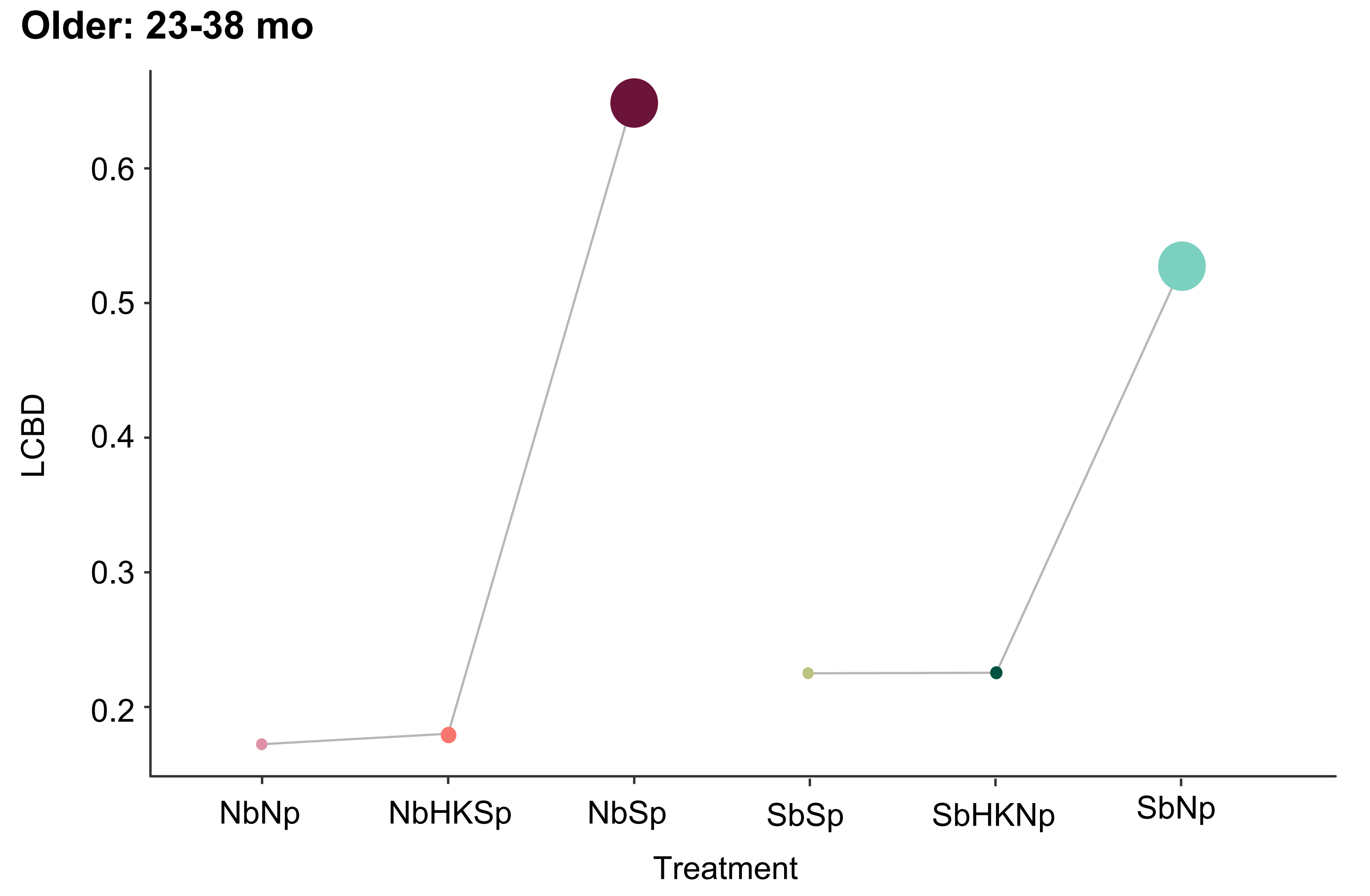
Older: 23-38 mo
LCBD
0.2
0.3
0.4
0.5
0.6
NbNp
NbHKSp
NbSp
SbSp
SbHKNp
SbNp
Treatment

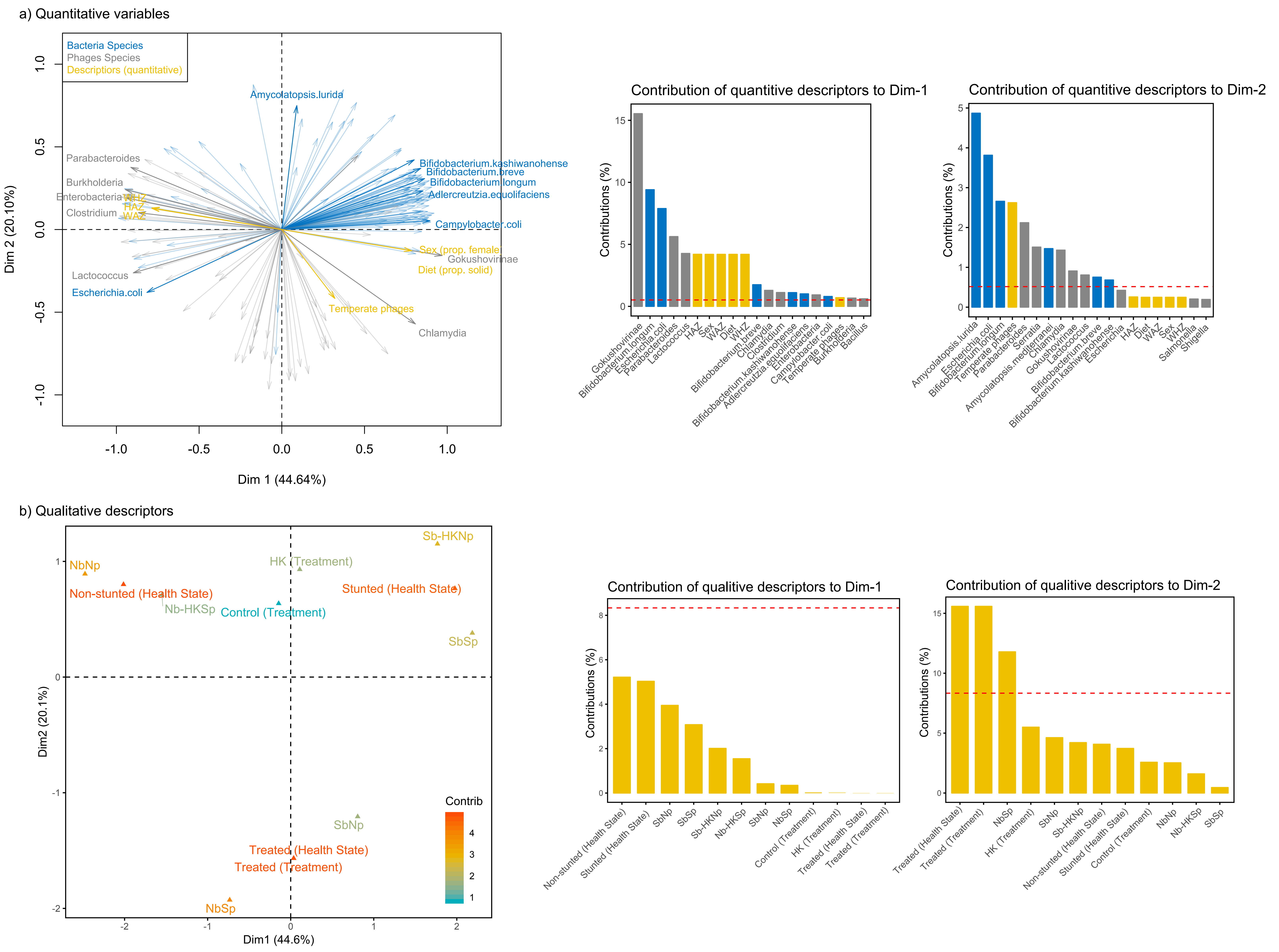
a) Quantitative variables
Bacteria Species
Phages Species
Descriptors (quantitative)
Amycolatopsis.lurida
Parabacteroides
Burkholderia
Enterobacteria
Clostridium
WHZ
HAZ
WAZ
Bifidobacterium.kashiwanohense
Bifidobacterium.breve
Bifidobacterium.longum
Adlercreutzia.equolifaciens
Campylobacter.coli
Sex (prop. female)
Gokushovirinae
Diet (prop. solid)
Lactococcus
Escherichia.coli
Temperate phages
Chlamydia
Dim 1 (44.64%)
Dim 2 (20.10%)
Contribution of quantitive descriptors to Dim-1
Contribution of quantitive descriptors to Dim-2
Contributions (%)
b) Qualitative descriptors
Sb-HKNp
NbNp
HK (Treatment)
Non-stunted (Health State)
Stunted (Health State)
Nb-HKSp
Control (Treatment)
SbSp
SbNp
Treated (Health State)
Treated (Treatment)
NbSp
Contrib
Dim1 (44.6%)
Dim2 (20.1%)
Contribution of qualitive descriptors to Dim-1
Contribution of qualitive descriptors to Dim-2
Contributions (%)

a) Quantitative variables

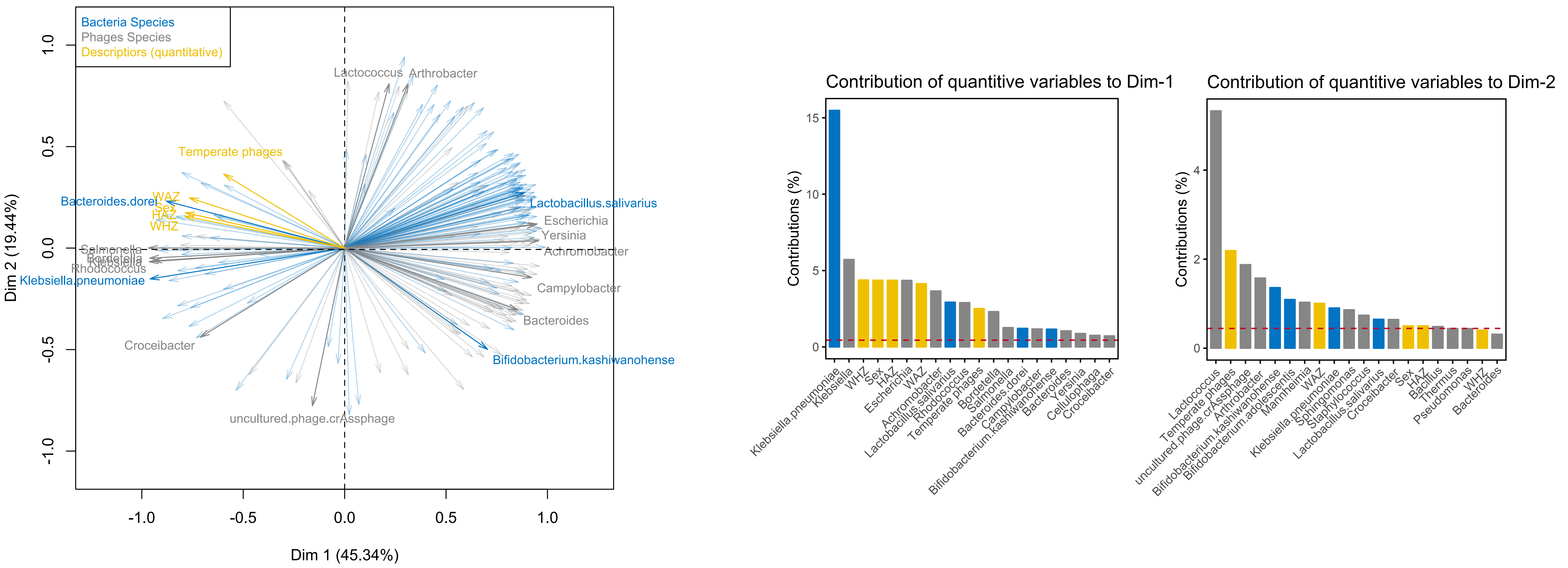

b) Qualitative descriptors

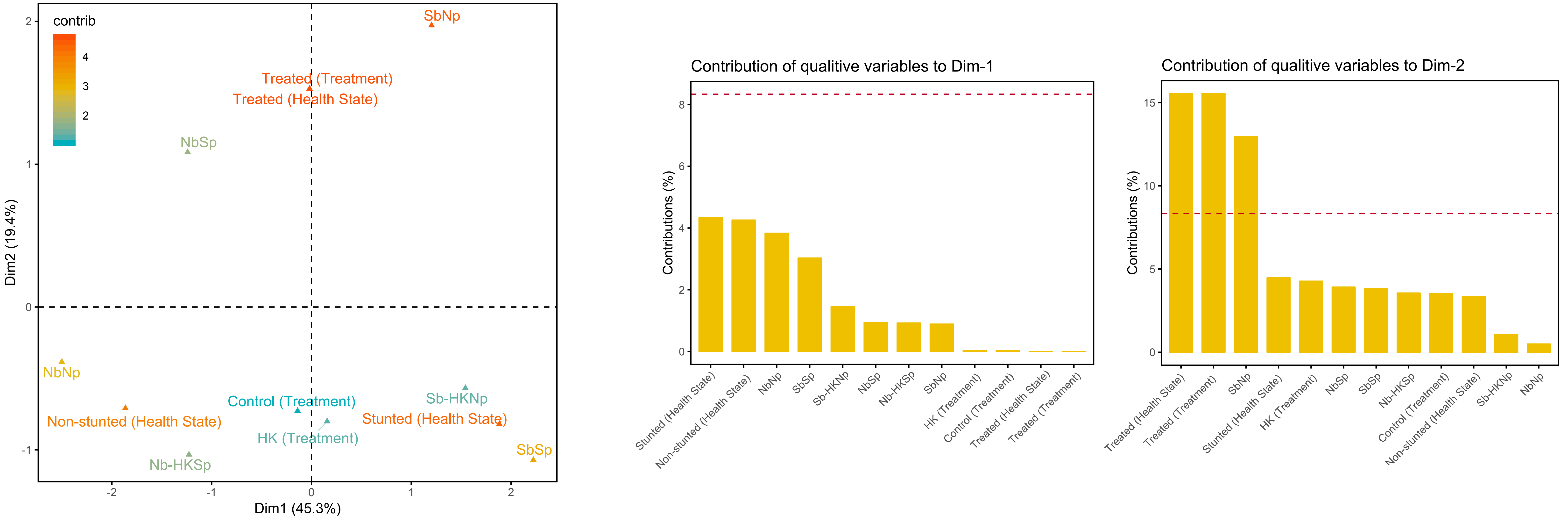

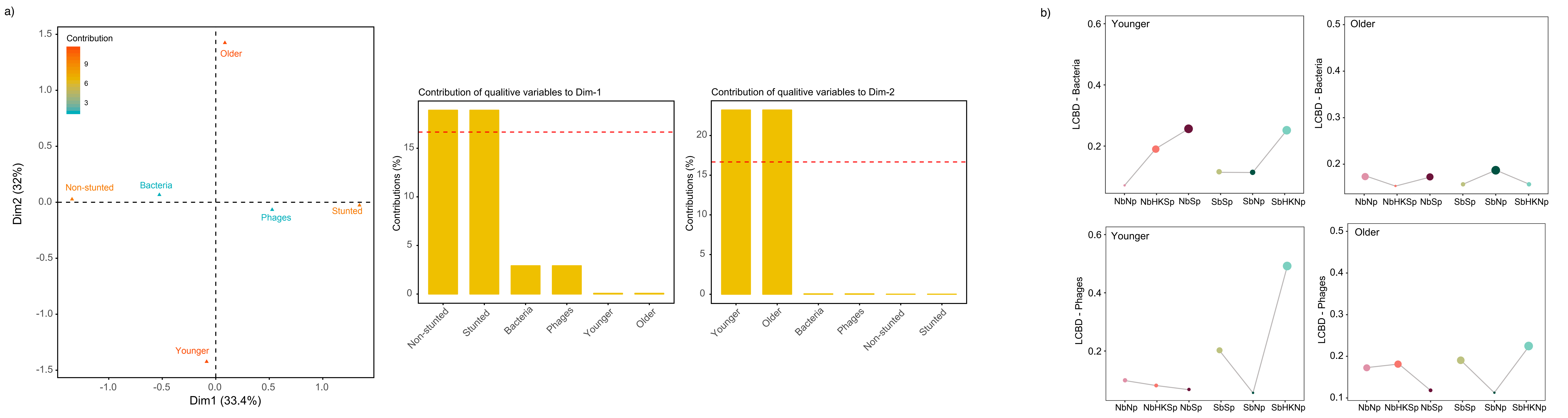
a)
Contribution
9
6
3
Older
Non-stunted
Bacteria
Phages
Stunted
Younger
Dim1 (33.4%)
Dim2 (32%)
Contribution of qualitive variables to Dim-1
Contributions (%)
Non-stunted
Stunted
Bacteria
Phages
Younger
Older
Contribution of qualitive variables to Dim-2
Contributions (%)
Younger
Older
Bacteria
Phages
Non-stunted
Stunted
b)
Younger
Older
LCBD - Bacteria
LCBD - Phages
NbNp
NbHKSp
NbSp
SbSp
SbNp
SbHKNp
