# Supplementary material for: Bacteriophages Isolated from Stunted Children Can Regulate Gut Bacterial Communities in an Age-Specific Manner
Source: Cell Host Microbe. 2020 Feb 12;27(2):199–212.e5. doi: 10.1016/j.chom.2020.01.004 (PMC7013830; doi:10.1016/j.chom.2020.01.004)
Supplement: Document S2. Article plus Supplemental Information [file mmc5.pdf]

Article

Cell Host & Microbe

Bacteriophages Isolated from Stunted Children Can Regulate Gut Bacterial Communities in an Age-Specific Manner

Graphical Abstract

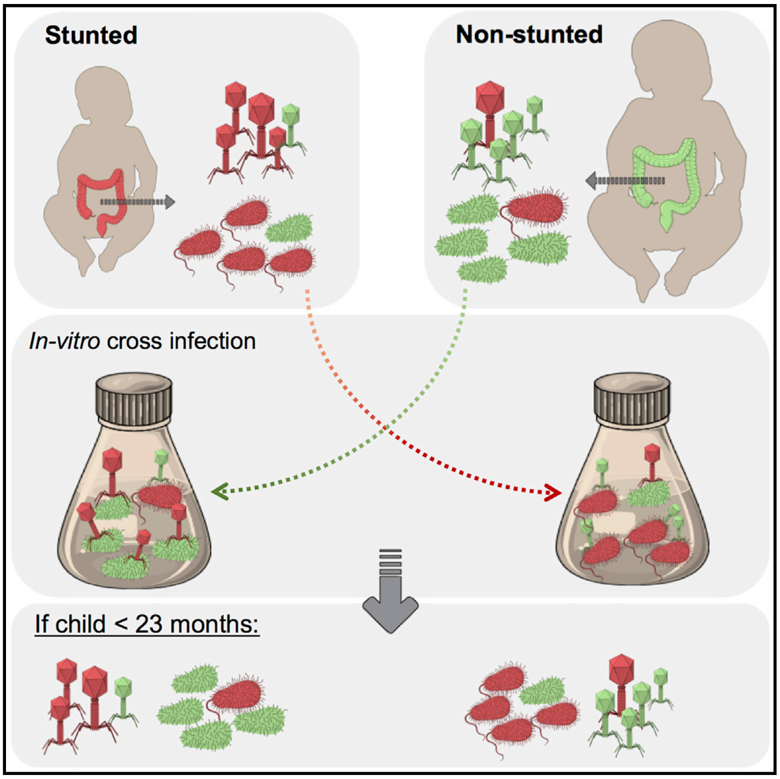

Highlights

- Stunted children have distinct gut phage communities relative to non-stunted ones
- *In vitro*, phages regulate bacteria isolated from children younger than 23 months
- Non-stunted children harbor more temperate phages, irrespective of age
- Bacteria in stunted children have more traits linked to human disease and metabolism

Authors

Mohammadali Khan Mirzaei, Md. Anik Ashfaq Khan, Prakash Ghosh, ..., Li Deng, Dinesh Mondal, Corinne F. Maurice

Correspondence

corinne.maurice@mcgill.ca

In Brief

Khan Mirzeai et al. describe the distinct gut bacteriophage communities of stunted and non-stunted young Bangladeshi children. *In vitro*, these bacteriophages regulate bacterial communities in an age-specific manner. This work highlights the potential of bacteriophages for changing the gut bacterial community in child stunting.

Khan Mirzaei et al., 2020, Cell Host & Microbe *27*, 199–212
February 12, 2020 © 2020 The Author(s). Published by Elsevier Inc.
https://doi.org/10.1016/j.chom.2020.01.004

CellPress

CellPress

Bacteriophages Isolated from Stunted Children Can Regulate Gut Bacterial Communities in an Age-Specific Manner

Mohammadali Khan Mirzaei,[1] Md. Anik Ashfaq Khan,[2] Prakash Ghosh,[2] Zofia E. Taranu,[3] Mariia Taguer,[1] Jinlong Ru,[4] Rajashree Chowdhury,[2] Md. Mamun Kabir,[5] Li Deng,[4] Dinesh Mondal,[2] and Corinne F. Maurice[1,6,*]
[1]Microbiology & Immunology Department, McGill University, Montreal, QC H3G 0B1, Canada
[2]Nutrition and Clinical Services Division, International Centre for Diarrheal Disease Research, Bangladesh (icddr,b), Dhaka 1212, Bangladesh
[3]Aquatic Contaminants Research Division (ACRD), Environment and Climate Change Canada (ECCC), Montréal, QC H2Y 2E7, Canada
[4]Institute of Virology, Helmholtz Centre Munich and Technical University of Munich, Neuherberg, Bavaria 85764, Germany
[5]Infectious Diseases Division, International Centre for Diarrheal Disease Research, Bangladesh (icddr,b), Dhaka 1212, Bangladesh
[6]Lead Contact
*Correspondence: corinne.maurice@mcgill.ca
https://doi.org/10.1016/j.chom.2020.01.004

SUMMARY

Stunting, a severe and multigenerational growth impairment, globally affects 22% of children under the age of 5 years. Stunted children have altered gut bacterial communities with higher proportions of Proteobacteria, a phylum with several known human pathogens. Despite the links between an altered gut microbiota and stunting, the role of bacteriophages, highly abundant bacterial viruses, is unknown. Here, we describe the gut bacterial and bacteriophage communities of Bangladeshi stunted children younger than 38 months. We show that these children harbor distinct gut bacteriophages relative to their non-stunted counterparts. *In vitro*, these gut bacteriophages are infectious and can regulate bacterial abundance and composition in an age-specific manner, highlighting their possible role in the pathophysiology of child stunting. Specifically, Proteobacteria from non-stunted children increased in the presence of phages from younger stunted children, suggesting that phages could contribute to the bacterial community changes observed in child stunting.

INTRODUCTION

Malnutrition is a major health concern in low- and middle-income countries and the leading cause of death in children younger than 5 years (Müller and Krawinkel, 2005). Child malnutrition falls under three main subtypes: underweight, stunting, and wasting. Stunting is defined by the World Health Organization (WHO) as a height-for-age *Z* score (HAZ) that is two standard deviations below the WHO Child Growth Standards mean or more (Lundeen et al., 2014). The onset of stunting occurs in children between the ages of 6 and 23 months old, when linear growth is highly susceptible to nutritional deficiency and environmental stress. This is also the transition period from exclusive milk feed to solid food. Most children from countries with high stunting rates do not have regular access to nutrient-rich or nutrient-balanced foods, which emphasizes the role of diet in child stunting (Menon et al., 2015). In addition to nutritional deficiencies, stunting is linked to repeated diarrheal infections and poor sanitation (MAL-ED Network Investigators et al., 2017). This multifactorial condition has multigenerational consequences and is associated with increased susceptibility to chronic diseases, poor education performance, and poor socioeconomic conditions (Blanton et al., 2016; Vonaesch et al., 2018). Although the health risks and environmental drivers of child stunting are well documented, the underlying pathophysiological mechanisms for this disease remain largely unknown, and recent studies have unveiled a correlation between an altered gut microbiota and stunting (Million et al., 2017; Vonaesch et al., 2018).

The gut microbiota refers to the community of microorganisms living in the gastrointestinal tract, including bacteria and bacteriophages (or phages). The gut microbiota plays essential roles in host metabolism, immune modulation, and colonization resistance to pathogens. In healthy individuals, approximately 90% of the gut bacterial community is dominated by members of the Firmicutes and Bacteroides phyla, whereas members of the Proteobacteria, containing many pathogens, reside at low levels (Blanton et al., 2016; Vonaesch et al., 2018). Shifts in gut bacterial composition have been associated with increasing numbers of diseases including inflammatory bowel diseases (IBD), allergies, diabetes, and obesity (Blanton et al., 2016; Mirzaei and Maurice, 2017). Generally, an increase in members of the Proteobacteria and lower overall community diversity are observed, in addition to disease-specific shifts of the gut microbiota. Stunted children also harbor an altered gut microbiota, with increased prevalence of *Enterobacteriaceae*, which may further contribute to their impaired growth and nutritional deficiencies. Indeed, the local inflammation caused by overgrowth of *Enterobacteriaceae* has been shown to lead to impaired digestive and absorptive functions of the gut, all linked to stunting (Krajmalnik-Brown et al., 2012; Vonaesch et al., 2018). In addition, recent studies further report that stunted children have an immature

This is an open access article under the CC BY license (http://creativecommons.org/licenses/by/4.0/).

gut microbiota relative to their non-stunted age-matched counterparts. This immaturity was defined as a lower microbiota-for-age *Z* score, characterized by a lower α-diversity of the gut microbiota and disproportionately higher levels of Proteobacteria (Subramanian et al., 2014).

In contrast to the work done to date on gut bacterial communities, the role of gut phage communities in child stunting remains largely unexplored. Phages are bacterial viruses that are key to the maintenance and function of many ecosystems by supplying bacteria with genes involved in host adaptation, toxin production, and metabolism (Scanlan, 2017; Thompson et al., 2011). Phages control bacterial diversity and abundance and can modify the O-antigen component of lipopolysaccharide (LPS) in gram-negative bacteria, which is particularly relevant to intestinal inflammation (Van Belleghem et al., 2018). In the gut, phages are abundant, with a phage-to-bacteria ratio close to 1:1 based on sequencing data (Shkoporov and Hill, 2019). Phage communities are distinct between individuals and stable over time compared with gut bacterial ones, despite a few examples of common and widespread phages, such as crAssphage, and rapidly evolving phages (Minot et al., 2013; Yutin et al., 2018). Recently, changes in the diversity and abundance of phages have been associated with many diseases such as IBD, diabetes, malnutrition, AIDS, and Parkinson's disease, highlighting their potential role in human health (Duerkop et al., 2018; Ma et al., 2018; Minot et al., 2011; Norman et al., 2015; Tetz et al., 2018).

Despite the known effects of phages on bacterial communities, bacteria-phage dynamics in the gut remains poorly described (Džunková et al., 2019; Manrique et al., 2017; Shkoporov and Hill, 2019). This is an important limitation to our functional understanding of how the gut microbiota alters human health. Relevant examples of the narrow host range of phages and their effective therapeutic use provide compelling arguments that phages could be used to manipulate gut bacterial communities to promote health (Kortright et al., 2019; Scanlan, 2017). Here, we explored whole community bacteria-phage population dynamics from stunted and non-stunted, otherwise clinically healthy, children from Dhaka, Bangladesh. Despite the significant progress in tackling malnutrition, Bangladesh still has one of the highest rates of child stunting in the world, affecting an average of 36.1% of children younger than 5 years (Nisbett et al., 2017). We obtained fecal samples from 30 non-stunted and 30 stunted children from the Mirpur slum area, aged between 14 and 38 months, and separated the free phage and bacterial communities. Because diet is a major regulator of gut bacterial diversity, we split the children into two age groups based on their age-specific diet, with each group consisting of 15 non-stunted and 15 stunted children. Children in the younger group, aged between 14 and 23 months, had a mixed diet of milk and solid food, whereas the older children, aged between 28 and 38 months, had fully transitioned to solid food for more than 2 months. After characterizing the abundance and diversity of the phage and bacterial communities in the original fecal samples, we proceeded with *in vitro* 2 × 2 factorial cross-infections of both communities, where bacteria from non-stunted children were exposed to phages from stunted children and vice versa. This allowed us to (1) identify the differences in bacteria and phage communities between non-stunted and stunted children; (2) determine how phages modify gut bacterial communities of non-stunted and stunted children; and (3) investigate the role of the children's age, diet, and health conditions on bacteria-phage interactions.

RESULTS

Children Metadata

The younger age group of children comprised an equal number of boys and girls with a mean age of 19.5 ± 2.5 months, mean weight of 9.6 ± 1.3 kg, mean height of 77.7 ± 3.4 cm, and a mean HAZ score of −1.8 ± 0.9. Children in the older age group comprised 18 girls (60%) and 12 boys (40%), with a mean age of 33.2 ± 2.9 months, mean weight of 11.5 ± 0.9 kg, mean height of 87.4 ± 3.1 cm, and a mean HAZ of −1.7 ± 0.92 (Table S1). In younger children, 90% of them were fed breast milk; the rest received a mixture of breast milk and formula. Median HAZ scores of non-stunted children in the younger group (−0.97; IQR: −1.44 to −0.73) and the older age group (−1.27; IQR: −1.47 to −0.55) were significantly higher (Wilcoxon, matched-pairs signed rank test, $p < 0.0001$) than the median HAZ scores of the children with stunting in the younger age group (−2.53; IQR: −2.97 to −2.27) and the older stunted children (−2.32; IQR: −2.79 to −2.1), respectively.

Bacterial and Phage Abundances in Non-stunted and Stunted Children

To examine differences in bacterial and phage absolute abundances among all health status and age groups, we counted stained bacteria and viral-like particles (VLPs) by microscopy (see STAR Methods) (Figure S1A). There were no significant differences in bacterial or VLP abundances, irrespective of health status or age group (Figures S2A and S2B). Viral abundances in young children were more variable compared with the older ones, regardless of their health status (Figure S2A). This variability in VLP abundance is specific to young children and was not observed with the bacterial abundance data. We then determined the resulting virus-to-bacteria ratio (VBR) in all children. We report no statistical difference between the VBRs of the different groups, most probably because of the high variability in VLP abundance in the younger children (Figure S2C).

Bacterial Diversity in Non-stunted and Stunted Children

We used 16S rRNA gene and shotgun sequencing (Figure S1B; Table S3) to examine how bacterial community composition may differ according to health status and age group. We further examined how these changes in phylum or species composition affected the overall diversity by determining the Shannon Evenness Index, as well as bacterial richness of each age group. Actinobacteria were the most abundant in younger children regardless of health status, though their relative abundance was slightly higher in non-stunted children (Figure 1A). The second most common phylum in younger children was the Firmicutes. In contrast, in older children, the Firmicutes were the most abundant, followed by the Actinobacteria (Figure 1A). Stunted children carried more Proteobacteria compared with their non-stunted counterparts, with younger stunted children showing the highest proportion of Proteobacteria (9%; Figure 1A). At the species level, non-stunted younger children had more *Bifidobacterium adolescentis* (6%), whereas stunted children had a slightly greater proportion of *Bifidobacterium longum* (25%) and notably greater proportions of

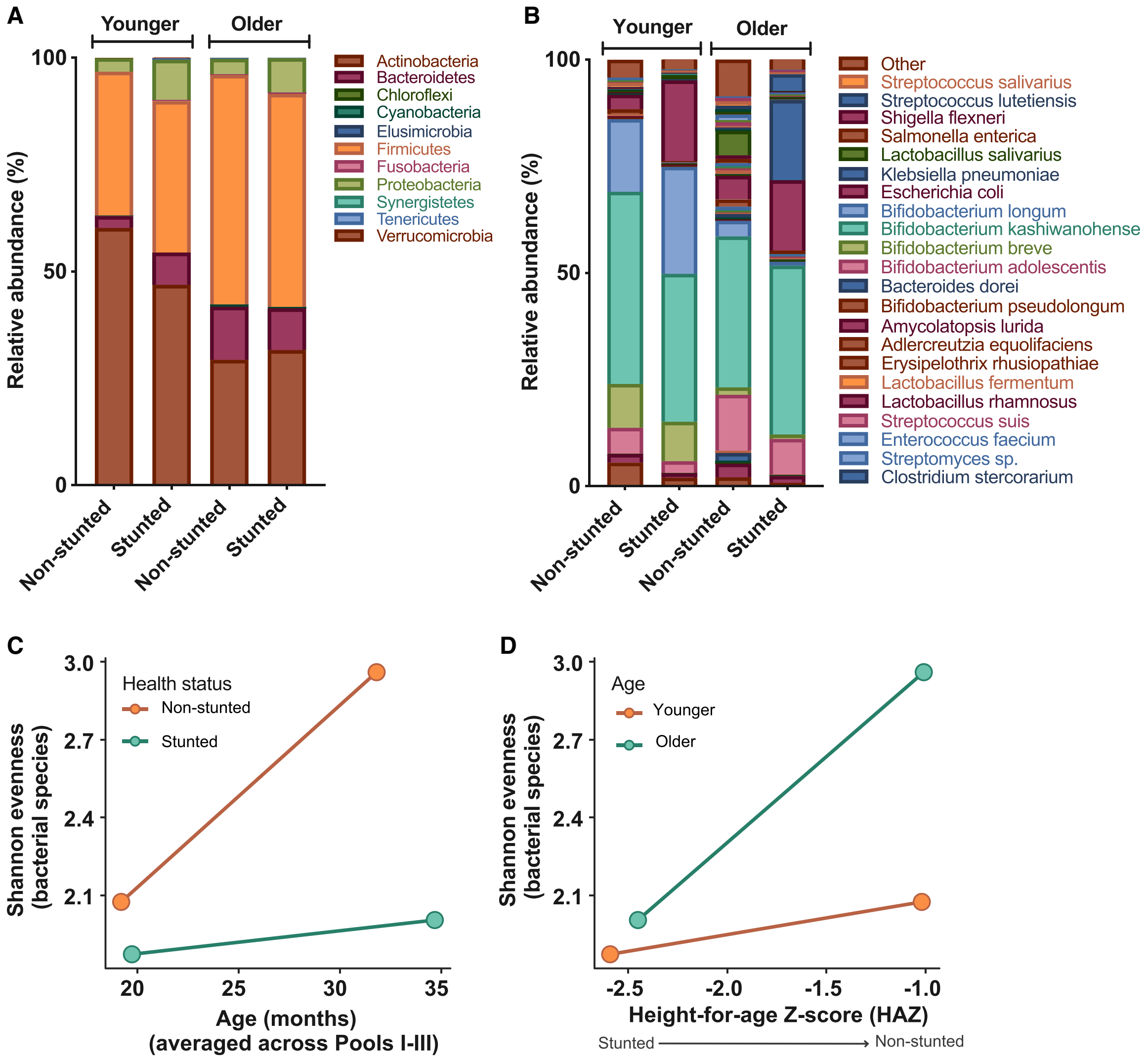

Figure 1. Bacterial Communities Are Less Diversified in Younger, Stunted Children

Relative abundance of bacterial phyla based on 16S analysis (A) and species based on shotgun metagenomics (B) in children's fecal samples (n = 60). Samples were pooled for sequencing (see STAR Methods). Children in the younger age group were aged between 14 and 23 months; children in the older age group were aged between 23 and 38 months. Shannon Diversity Index was compared among (C) age groups according to health status, and (D) HAZ scores according to age groups.

See also Figures S2 and S3.

Escherichia coli (19%) (Figure 1B). For older non-stunted children, we observed a greater proportion of *B. adolescentis* (increasing from 6% to 13%), as well as an increase in *Lactobacillus salivarius* (6%); stunted older children carried more *Klebsiella pneumoniae* (19%) and *E. coli* (16%) compared with their non-stunted counterparts. In general, the number of reads assigned to *B. longum* was greater in younger children than older ones, whereas *Bifidobacterium kashiwanohense* was the dominant species in all age and health groups (Figure 1B).

Though our sample size was too small to test for significance, we noted that the difference in bacterial species diversity and richness between non-stunted and stunted children tended to be greater for the older age group than the younger one (Figures 1C and 1D). This was due to a greater increase in diversity in non-stunted children with age. At the phyla level, diversity and richness only increased in the non-stunted group (Figure S3). Overall, this suggests that the effects of stunting became more apparent with age (Blanton et al., 2016; Dinh et al., 2016; Vonaesch et al., 2018).

Abundance of Proteobacteria Pathogenic Species

Given the suggested link between pathogenic bacteria in the gut and child stunting (Harper et al., 2018; Vonaesch et al., 2018), we analyzed the relative abundance of known human pathogenic species, focusing on Proteobacteria species (see STAR Methods). Within the Proteobacteria, *E. coli* was the most abundant potential pathogenic bacterial species in all age and health groups, except in older stunted children, where *Klebsiella pneumonia* dominated at 51% of the pathogenic community, followed by *E. coli* at 44% (Figure 2A). Young stunted children had the highest occurrence of *E. coli* (93% of reads within Proteobacteria)

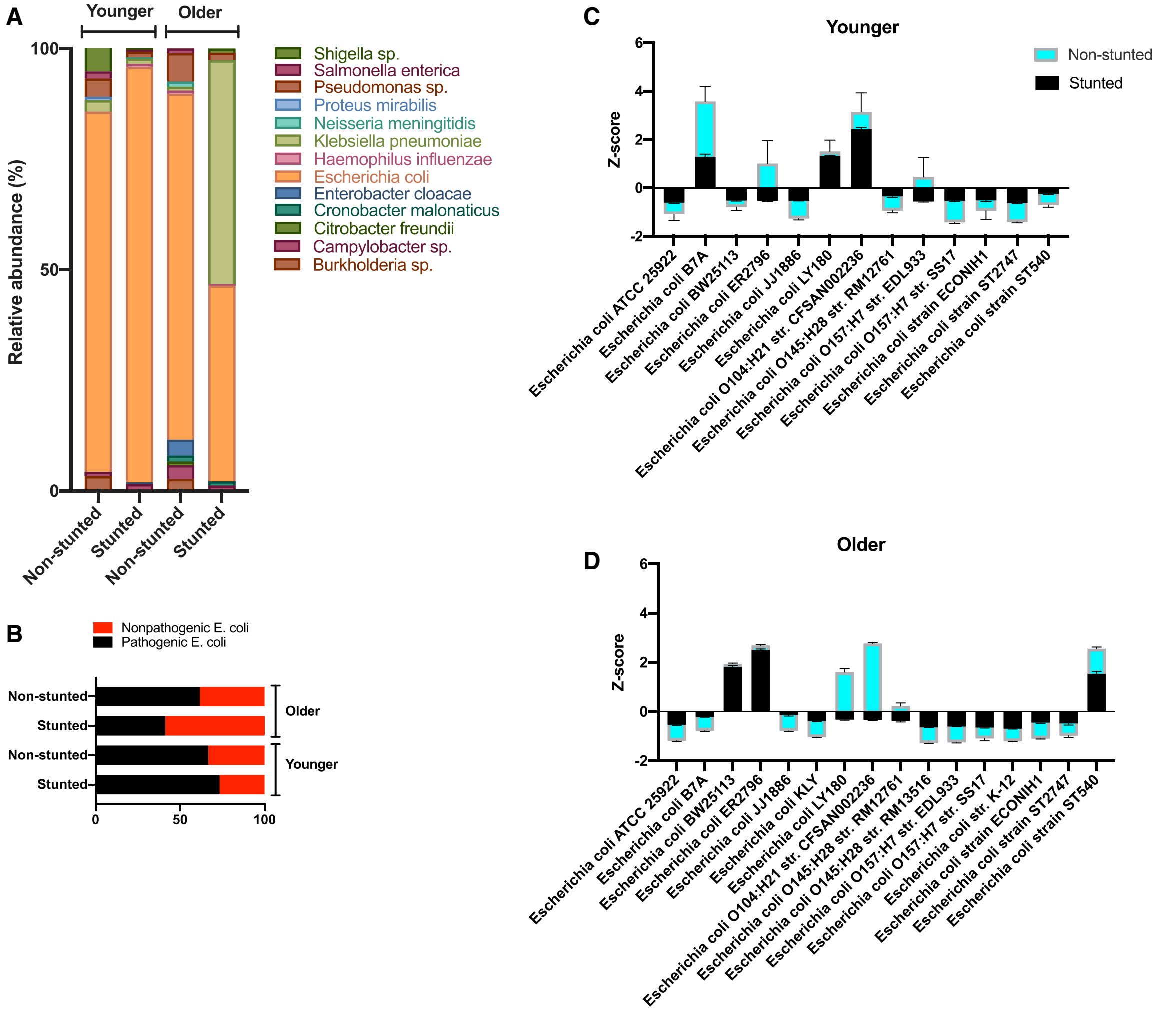

Figure 2. Non-stunted and Stunted Children Harbor Many Pathogenic *E. coli* Strains

(A) Relative abundance of known pathogenic species of Proteobacteria.

(B) Relative abundance of pathogenic and non-pathogenic *E. coli* strains.

(C and D) *Z* scores of the abundance of *E. coli* strains in children from the younger (C) and older (D) age groups. When present, error bars correspond to the standard deviation of bacterial abundances.

overall. We further analyzed the presence of pathogenic and non-pathogenic *E. coli* strains based on their nucleotide identity to known *E. coli* pathogenic strains (Table S2). The ratio of pathogenic to non-pathogenic strains of *E. coli* was higher for younger children (Figure 2B). Older stunted children had a relatively lower abundance of *E. coli*, likely due to competition with *K. pneumonia*, which dominated this age group (Juarez and Galvan, 2018). *E. coli* BW25113 and *E. coli* ER2796 were the dominant strains in older stunted children (Kruskal-Wallis, Dunn's post hoc test, $p < 0.05$), whereas *E. coli* LY180 and *E. coli* O104:H21 strain CFSAN002236 were the major *E. coli* strains in their non-stunted counterparts (one-way analysis of variance [ANOVA], Holm-Sidak's post hoc test, $p < 0.05$) (Figures 2C and 2D).

Phage Diversity in Non-stunted and Stunted Children

The majority of reads we identified as viruses were phages (94% on average); reads identified as nonbacterial viruses varied from 0.5% for younger stunted children to 14% for older non-stunted children. Nonbacterial viruses were archaeal, human, and environmental viruses including amoeba and algae viruses. The proportion of nonbacterial viruses was highest for non-stunted older children, with most reads belonging to Circoviruses, a DNA virus commonly found in humans (Li et al., 2010). On average, 36% of the reads could not be assigned to any reference phage genomes.

Members of the Siphoviridae family dominated phage reads in all children (Figure 3A). Young, non-stunted children had high proportions of Podoviridae (28%), Myoviridae (14%), and Microviridae (6%); whereas older, non-stunted children had a greater proportion of reads assigned to Myoviridae (21%) and Microviridae (18%). In the stunted group, young children had less Podoviridae (12%) than older ones (23%). At the species level, *Bacillus* phages were the most abundant phages in young, non-stunted children, totaling 14% of the reads (Figure 3B), followed by crAssphage at 7%. Young, stunted children carried

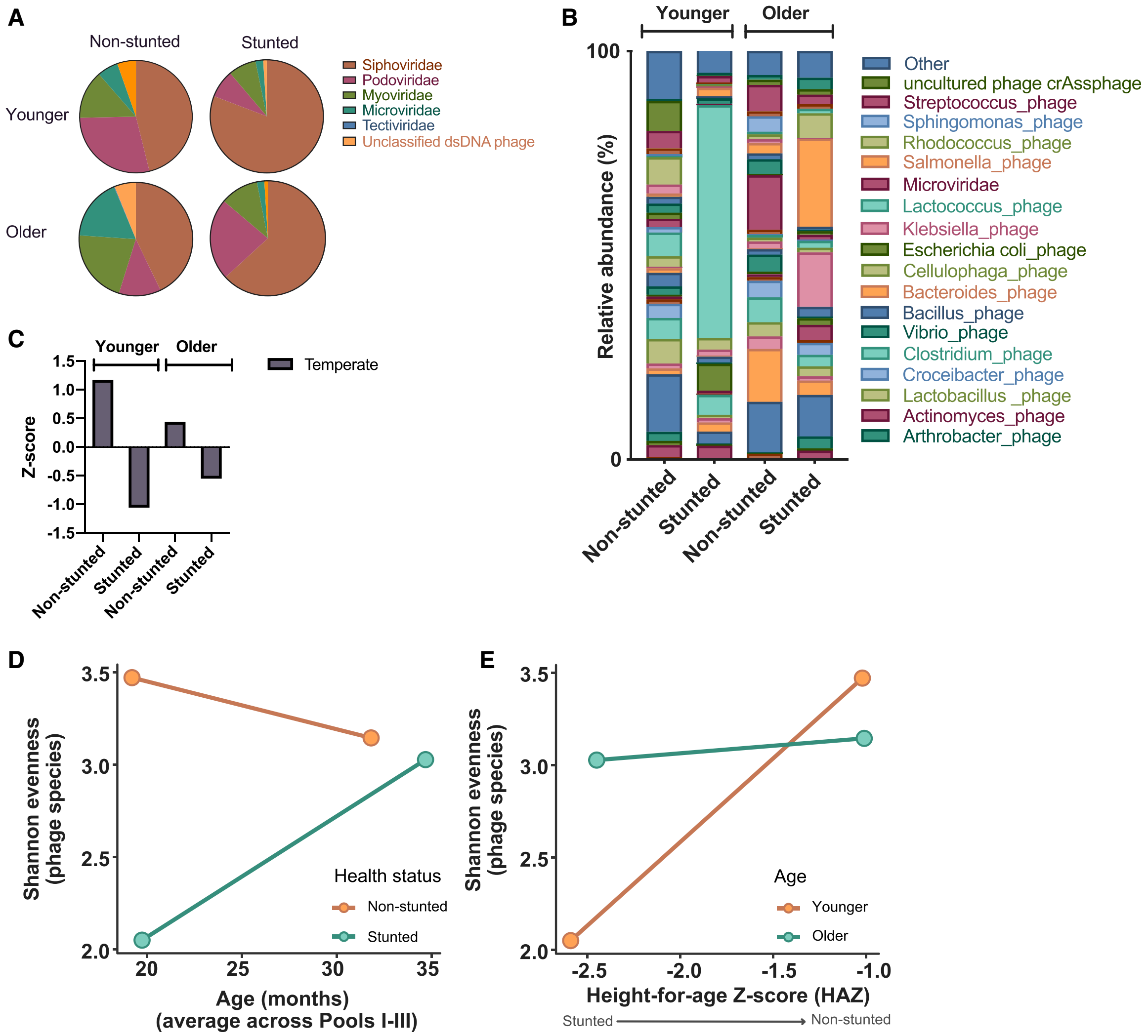

Figure 3. Phage Community Composition and Diversity Indices Change with Age and Health

Relative abundance of phage species in all fecal samples. Samples were pooled for sequencing (see STAR Methods). Relative abundance of (A) phage families, (B) phage species, and (C) abundance of temperate phages presented as *Z* score. Shannon diversity in fecal samples of children was compared among (D) age groups according to health status, and (E) HAZ scores according to age groups.

See also Figure S2.

more *Lactococcus* phages (56%), followed by *E. coli* phages (6%). In contrast, Microviridae and *Bacteroides* phages were the most abundant phages in older, non-stunted children (both at 13%), closely followed by *Bacillus* phages (12%), whereas older, stunted children had more *Salmonella* (21%) and *Klebsiella* (13%) phages. In terms of diversity and richness, phage species followed a pattern similar to that of bacteria, with a tendency for higher diversity in non-stunted children compared with stunted ones (Figures 3D and 3E). Contrasting with the bacterial data, the difference in phage diversity between non-stunted and stunted children tended to decrease with age (Figure 3D).

We next aimed to determine the dominant phage replication cycle, as the effects of phages can vary based on their replication cycle (lytic versus lysogenic cycle) (Mirzaei and Maurice, 2017). Most temperate phages integrate their genome into their bacterial host, thus protecting their host against further infection by closely related phages, and potentially transferring genes providing a competitive advantage (Shkoporov and Hill, 2019). We identified temperate phages based on the presence of the *integrase* or *integrase*-like genes, which are used by most known temperate phages (see STAR Methods). On average, young children had more temperate phages than older children, irrespective of health status; however, within each age group, non-stunted children had more temperate phages than their stunted counterparts (young: non-stunted *Z* score = 1.2 and stunted *Z* = −1; old: non-stunted *Z* = 0.4 and stunted *Z* = −0.5) (Figure 3C).

Bacterial and VLP Abundances and Diversity after Cross-Infections

To gain a whole community perspective on bacteria-phage interactions, and to test if gut phages isolated from non-stunted and stunted children alter gut bacterial communities from children

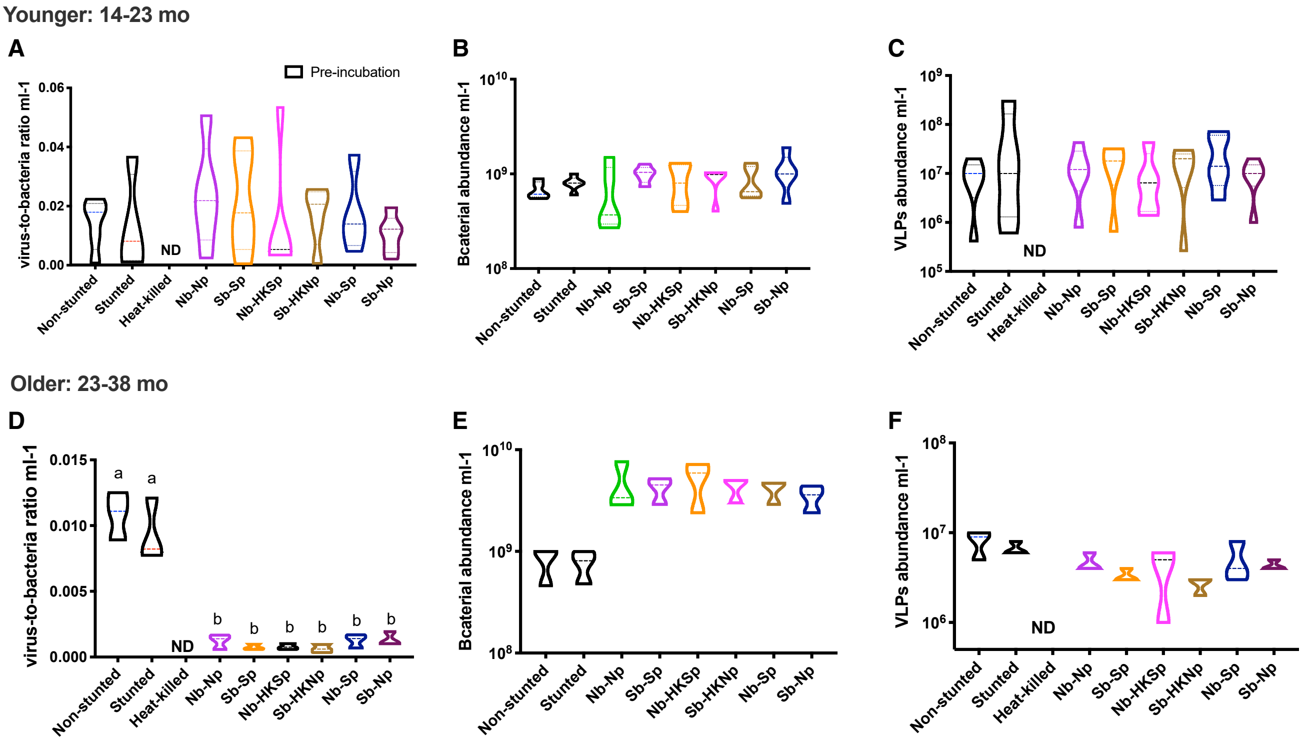

Figure 4. Virus-to-Bacteria Ratios Change after Cross-Infections in an Age-Specific Manner

Violin plots of whole community bacterial and VLP abundances in children from the younger (A–C) and older (D–F) age groups prior and post cross-infections. (A and D) Virus-to-bacteria ratios; (B and E) bacterial abundances by epifluorescence microscopy; (C and F) abundance of VLPs by epifluorescence microscopy. Bars connected by the same letter are not significantly different ($p < 0.05$, one-way ANOVA, Holm-Sidak's multiple comparisons test). ND, not detected; N, non-stunted; S, stunted; b, bacteria; p, phage; HK, heat killed. The width of the plots reflects the data frequency distribution; dotted lines show the median and lower and upper quartiles.

with different health statuses, we proceeded with fully factorial cross-infection experiments in each age group (Figure S1). We then determined bacterial and viral absolute abundances (Figure 4), as well as bacterial and phage community composition. While the VBRs in younger children remained similar between the start and the end of the cross-infections (Figure 4A), we noted a significant decrease in VBRs in older children between the original fecal samples and the cross-infected cohorts (Figure 4D; $p < 0.05$, one-way ANOVA, Holm-Sidak's multiple comparisons test). This change was mostly driven by an increase in bacterial abundances during incubation (Figures 4E and 4F).

The larger sample size for the cross-infections allowed us to test for significant differences in diversity and richness among cohorts. We found that at the phyla level, diversity was significantly greater in cross-infected cohorts of younger children relative to older children (Figure S4A; $t = 7.4$, $p < 0.00001$). Bacterial phyla richness was greater in older stunted children than older non-stunted children ($t = -7.78$, $p = 0.001$) after cross-infections (Figure S4B), whereas younger stunted and non-stunted children had similar richness ($t = 0$, $p = 1$). When bacteria from non-stunted children (Nb) were exposed to phages from stunted children (Sp), Proteobacteria (mainly *Enterobacteriaceae*) increased by 6% relative to the heat-killed control (Nb-HKSp) and nearly doubled from the incubation control (NbNp) in younger children (treatment: 6,719.6 ± 8,818.7 reads; heat-killed control: 3,847 ± 3,659.1 reads; incubation control: 3,223.6 ± 2,827.9 reads) (Figure 5A). In contrast, Proteobacteria decreased when bacteria from stunted children (Sb) were exposed to phages from non-stunted children (Np). This did not seem to be driven by the phages themselves, as the difference between live phages or heat-killed phages was small (treatment: 2,279 ± 1,609.7 reads; heat-killed: 1,621.6 ± 2,315.3 reads; incubation control: 6,223 ± 8,054 reads). A similar trend was observed for Firmicutes (mostly *Veillonellaceae*) when bacteria from stunted children were exposed to phages from non-stunted children: the relative abundance of Firmicutes increased compared with that of the controls (treatment 11,650.6 ± 8,396.1 reads; heat-killed: 5,688 ± 3,680.4 reads; incubation control: 6,546 ± 3,386.9 reads). Such changes in dominant phyla were not observed in older children.

Bacteria and Phage Species Turnover after Cross-Infection

To identify which species varied the most among the cross-infection treatments, we quantified the contributions of each species to β-diversity (or species turnover; calculated here as the species contribution to β-diversity [SCBD]) for both the bacteria and phage species. Similarly, to test which treatment had the most unique community composition and greatest turnover relative to other treatments, we calculated the contribution of each treatment to the total β-diversity (or local contribution to β-diversity [LCBD]). For the bacterial species of younger

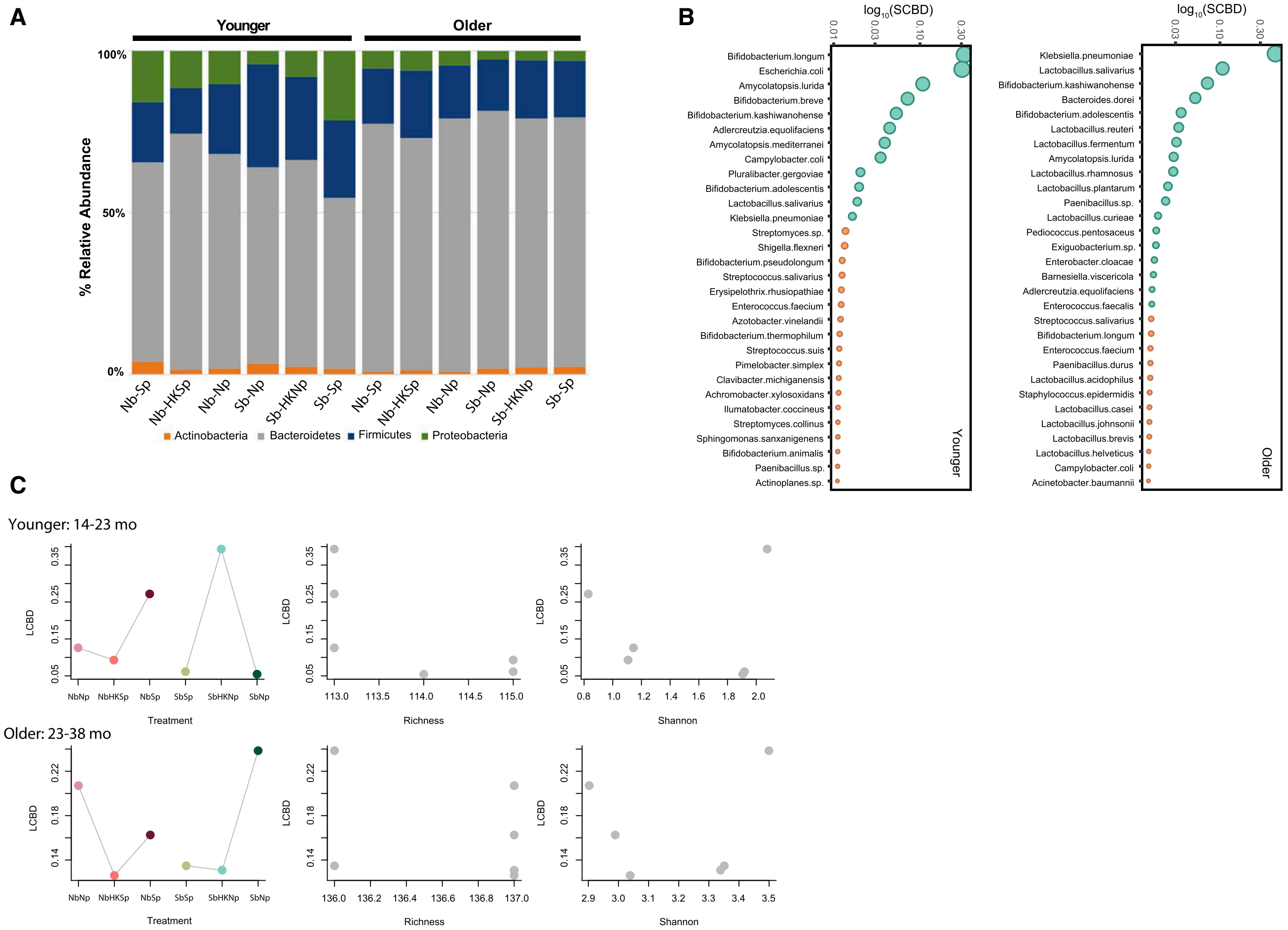

Figure 5. Phage Communities Alter Bacterial Diversity in an Age-Specific Manner

(A) Relative abundance of bacterial phyla after cross-infections in both age groups

(B) Bacterial species contribution to β-diversity (SCBD) in younger (left) and older (right) children. Shown are the top 30 species with greatest SCBD values for each age group, where green points correspond to species with SCBD values greater than the mean SCBD values of all species (n = 179 species total).

(C) Relationship between local contribution of samples to bacterial β-diversity (LCBD) and diversity indices after cross-infections for the younger (top) and the older age groups (bottom). Graphs 1 and 4 correspond to LCBD versus treatment type, where cross-infection samples with non-stunted bacteria are shown in red, and those with stunted bacteria are in green. Graphs 2 and 5 correspond to LCBD versus species richness. Graphs 3 and 6 correspond to LCBD versus Shannon evenness.

See also Figures S4 and S5.

children, the only treatment with a significant LCBD was bacteria from non-stunted children (Nb) exposed to phages from stunted children (Sp) relative to their controls. This treatment also had the lowest bacterial Shannon Diversity Index compared with its controls (Figure 5C). The species that contributed the most to change was *Amycolatopsis lurida* (highest species contribution to β-diversity; Figure 5B). When bacteria from young stunted children (Sb) were infected with phages from young non-stunted children (Np), we found that the heat-killed controls had a significant LCBD and highest Shannon Diversity, but the lowest richness (thus they were unique because of a loss in species dominance; Figure 5C). *E. coli* showed the highest SCBD score, followed by *B. longum* (Figure 5B).

For the phage species of younger children, similar to the bacteria, the treatment with a significant LCBD was the bacterial communities from non-stunted children exposed to phages from stunted children (Figure S5). Contrasting with our bacterial results, this treatment did not have the lowest evenness among the treatment groups. In terms of species contributing the most to this change, we found that the Gokushovirinae, a ssDNA phage associated mostly with Gammaproteobacteria (Roux et al., 2012), had the highest contribution to β-diversity, followed by *Parabacteroides* phages.

The changes in the older children were limited and did not follow those of young children. For the bacterial species, there were no treatments with a significant LCBD (Figure 5C). However, for the phage communities, both treatments showed a significant change (LCBD) relative to their respective controls (Figure S5). The *Sphingomonas* phages contributed the most to species turnover when bacteria from non-stunted children were infected by phages from stunted children, whereas *Lactococcus* phages had the highest score when bacteria from

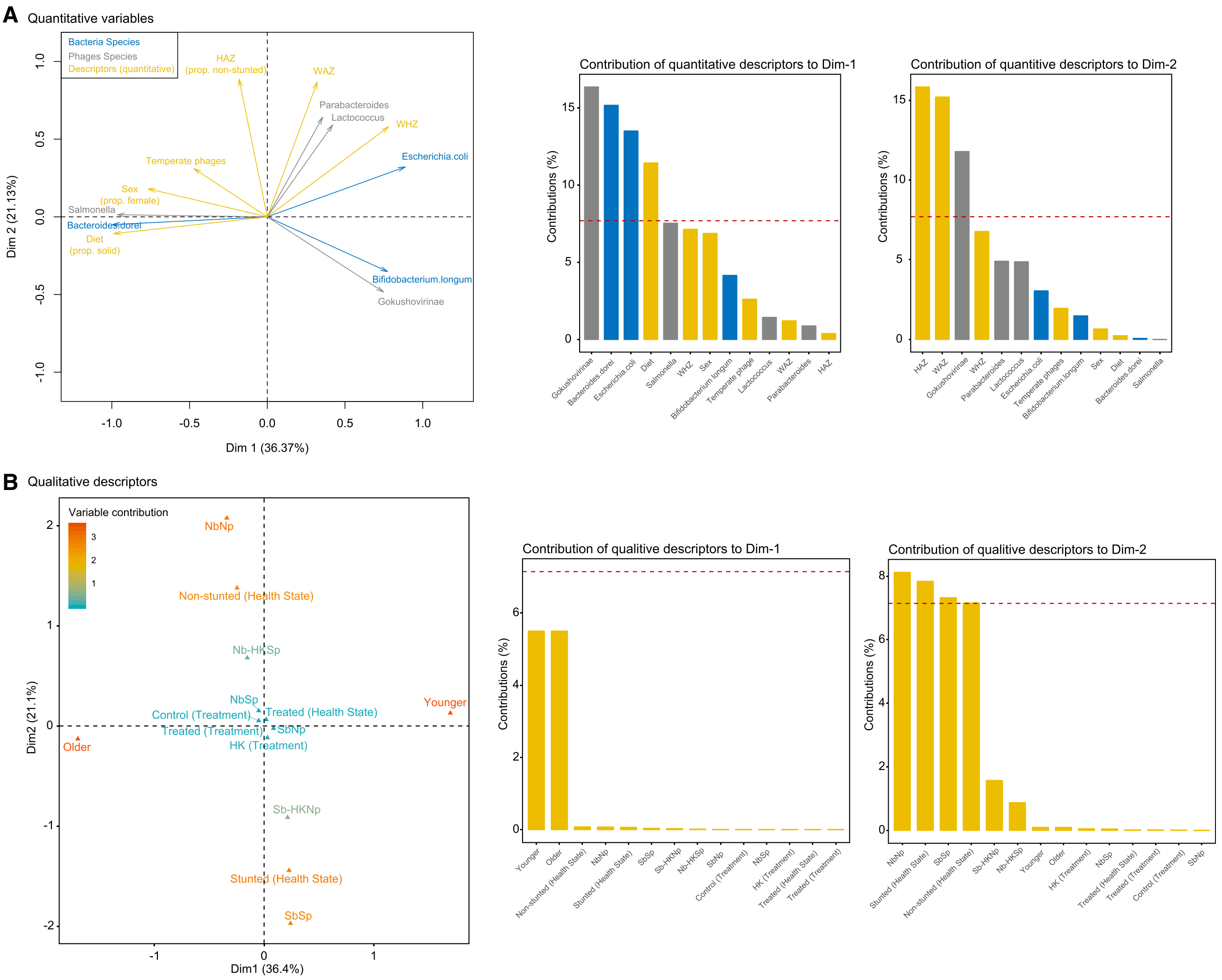

Figure 6. Multiple Factor Analysis of the Dominant Bacterial Species, Phage Species, Phage Replication Cycle, and Children Metadata for Both Age Groups

Dominant bacterial and phage species shown were selected using a PCA and the contribution circle from the cleanplot.pca() function.

(A) MFA ordination of quantitative variables, with the contribution of each qualitative variable to dimensions 1 and 2 of the MFA as insets.

(B) MFA ordination of qualitative variables. Quantitative variables include milk feed, age, sex, HAZ, WAZ, and WHZ; qualitative variables include health status, treatment type, and treatment factor. The Hellinger transformation of the bacterial and phage data, and the standardized values for the temperate replication cycle are presented. Quantitative children metadata was scaled.

See also Figures S6 and S7.

stunted children were infected with phages from non-stunted children. Furthermore, the pathogenic-nonpathogenic *E. coli* ratio did not change after cross-infections specific to different treatments within age groups (not shown).

Multiple Factor Analysis of the Interactions between Phages, Bacteria, and the Metadata

To synthesize the cross-infection results, we examined the correlations among the dominant bacterial and phage species, and the metadata (diet, age, sex, health status, cross-infection, HAZ, WAZ [weight-for-age Z], and WHZ [weight-for-height Z] of children) using a multiple factor analysis (MFA) framework (Borcard et al., 2018). This multivariate correlation allowed us to assess the contribution of each metadata variable to changes in the bacterial and phage populations. The first dimension of the MFA explained 36.37% of the total variation in the phage and bacterial community and descriptor variables (Figure 6A), and separated variables according to their correlation with age (Figure 6B). The second dimension of the MFA explained an additional 21.13% of the total variability in all three data matrices and largely separated variables correlated to non-stunted versus stunted children (Figure 6B). In light of these two axes (axis 1: age gradient, axis 2: health status gradient), we found that among the dominant bacteria and phage species, certain species tended to be more prominent in older children (i.e., *Salmonella* phage and *Bacteroides dorei*), whereas others were more prominent in the younger children (i.e., Gokushovirinae phage and *E. coli* and *B. longum*; Figure 6A). The Salmonella phage was also

associated with the temperate phage group. Finally, certain phage taxa (*Lactococcus* and *Parabacteroides*) were more common in non-stunted children, whereas Gokushovirinae were more prominent in stunted children after cross-infections.

We also ran MFAs to examine changes in phage and bacterial communities within each age group (Figures S6 and S7), this time examining all phage and bacterial species. In each case, the first two dimensions of the MFAs explained a large portion of the total variance in both communities and descriptor variables (>60%). In young children, the first dimension separated the non-stunted children with predominantly *Parabacteroides*, *Clostridium*, *Enterobacteria*, *Burkholderia*, and *Lactococcus* phages, and *E. coli*, from the stunted ones who had greater proportions of Gokushovirinae, *Chlamydia* phages, and numerous *Bifidobacterium* (Figure S6). The second dimension of the MFA separated heat-killed controls from treatments. Heat-killed controls were more strongly associated with temperate phages. In older children (Figure S7), the first dimension once again separated the non-stunted from stunted children, and the second dimension separated heat-killed from treated samples. As with the younger age group, the heat-killed control was associated with temperate phages. We also found that the bacterial and phage species associated either with non-stunted (*Bacteroides dorei*, *K. pneumoniae*, and the *Rhodococcus*, *Bordetella*, *Klebsiella*, and *Salmonella* phages) or stunted children differed from those identified in younger children, as noted in Figure 6.

MFA of the Interactions between Functional Traits and Metadata

Our last set of analyses examined changes in functional traits within original samples and within cross-infected cohorts. In line with previous reports (Minot et al., 2011; Moreno-Gallego et al., 2019), most metagenome reads (70% on average) mapped to genes with unknown function. In the original stool samples, we determined which Kyoto Encyclopedia of Genes and Genomes (KEGG) pathway was most abundant in each age and health group for both phages and bacteria. To evaluate this, we conducted an MFA correlating the functional trait matrix (expressed as relative abundances to normalize for the total number of reads per sample) with metadata. We only examined the dominant functional traits and removed the unknown traits to simplify the interpretation of the MFA (Figure 7). As before, we found that at the onset (original stool samples), non-stunted children had higher HAZ and WAZ (weight-per-age) scores than their stunted counterparts, whereas only young, non-stunted children had higher WHZ (weight-per-height) scores relative to other age and health groups (Figures 7 and S8). The MFA showed that young, stunted children had a greater number of traits related to human disease and metabolism, as well as traits related to cell growth and/or death and genetic replication and/or repair compared with non-stunted, older children (Figure 7). We note however that other (undetermined) pathways related to genetic replication and/or repair were likewise correlated to older children, which in turn were correlated to more solid diet and females. For the cross-infection samples, we were interested in identifying more specific changes in KEGG pathway abundances across treatments relative to their controls. We thus examined the turnover in pathway composition among cohorts, that is, the contribution of treatments (LCBD) and pathways (SCBD) to β-diversity. For the latter, we arbitrarily present the top 30 traits with highest contribution to β-diversity (Figure 7). In general, traits were most variable among treatments in younger children (Figure S8). Traits that contributed most to β-diversity in the younger age group were related to human disease, cellular process, and membrane transport, whereas those contributing most to β-diversity in the older age group were cellular process, signal transduction, carbohydrate metabolism, and xenobiotics biodegradation and/or metabolism (Figure S8).

DISCUSSION

Child stunting affects 22% of children under the age of 5 years worldwide, with long-term consequences on child development and higher risks for several chronic diseases (Müller and Krawinkel, 2005). Stunting is a multifactorial disease linked to nutritional deficiency, repeated diarrheal infections, poor sanitation, and altered gut bacterial communities (Blanton et al., 2016; Vonaesch et al., 2018). As phages are key to the maintenance and function of many ecosystems via their regulation of bacterial hosts, we hypothesized they could play a critical role in controlling gut bacterial communities in stunting (Džunková et al., 2019; Shkoporov and Hill, 2019). Through a combination of microscopy, 16S rRNA gene sequencing, and metagenomics, we characterized the fecal viral and bacterial communities of Bangladeshi children aged between 14 and 38 months to determine if phage communities differed between non-stunted and stunted children (or undergoing stunting), and identify how phages modify gut bacterial communities of non-stunted and stunted children at a whole community level. We also examined the relative importance of the children's age, diet, and health conditions on bacteria-phage interactions.

Consistent with previous reports, our data indicate high abundances of Proteobacteria and higher abundances of Actinobacteria than expected for their age among the enrolled children, resembling the gut bacterial composition in children younger than 1 year (Koenig et al., 2011). Actinobacteria and Proteobacteria are replaced during child development by Firmicutes and Bacteroidetes following the dietary transition from milk to solid food (Monira et al., 2011; Robertson et al., 2019). Thus, the higher-than-expected levels of Actinobacteria relative to the children's age could suggest a delay in microbiome development and/or maturity in these children. However, these high levels could also be partially explained by the higher prevalence of formula milk in the older children (Koenig et al., 2011; Ottman et al., 2012). Despite higher levels of Proteobacteria in stunted children relative to their non-stunted counterparts, our values are lower than those previously reported in these settings (Monira et al., 2011; Ottman et al., 2012). Nevertheless, the higher abundances of *Enterobacteriaceae* we observed, known to induce high and persistent levels of intestinal inflammation, coincide with overall lower abundance of Bacteroidetes, widely recognized for their ability to digest complex dietary compounds and improve energy harvested from food (Budge et al., 2019; Milani et al., 2017). The higher abundance of *K. pneumoniae* in older stunted children may suggest a role for this taxon in child stunting in this age group. Furthermore, the differences between these age groups were also observed through the distinct bacterial functional traits. Thus,

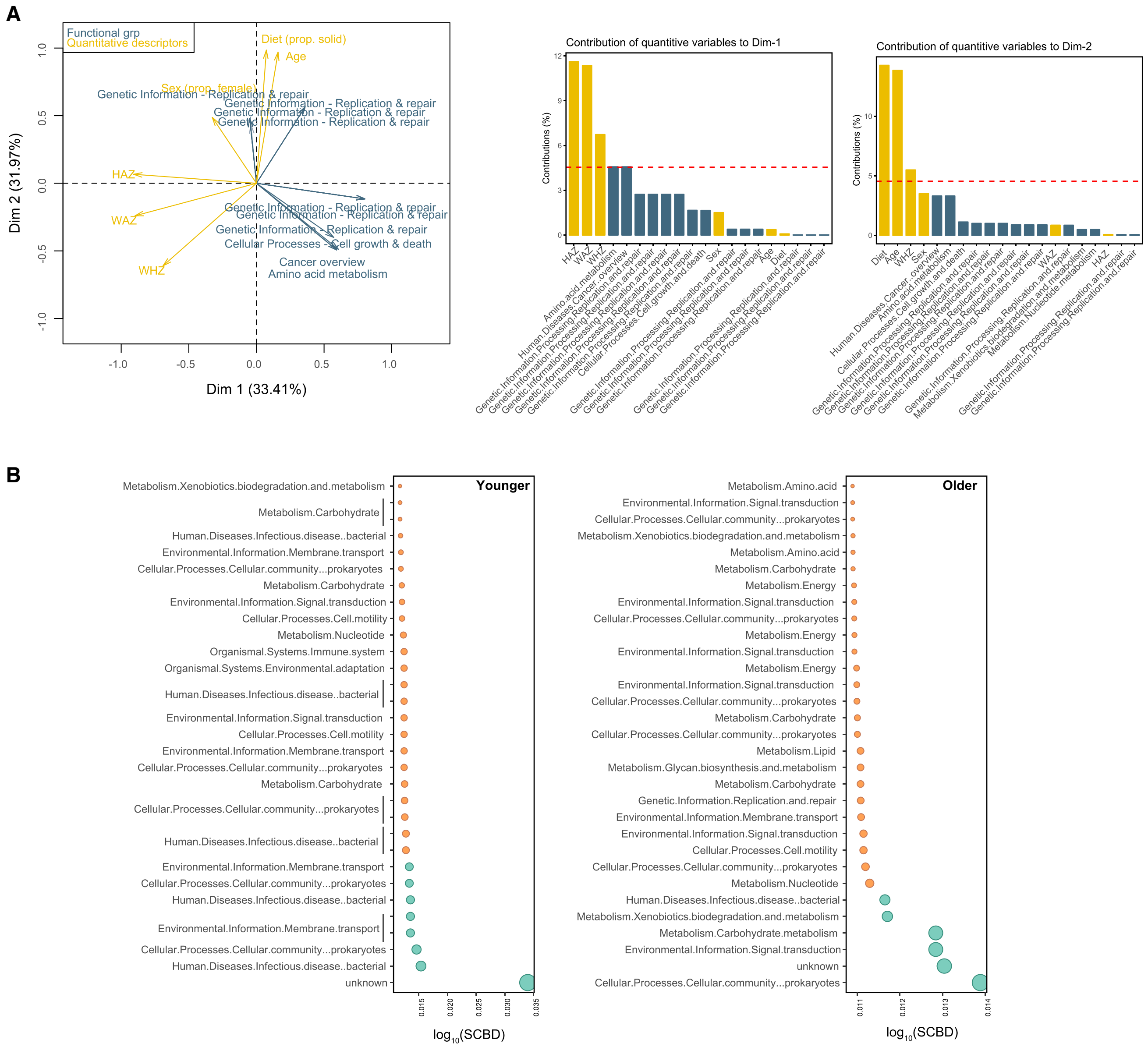

Figure 7. MFA of the Interactions between Bacterial Functional Traits and Metadata

(A) MFA ordination of dominant quantitative variables, with the contribution of each quantitative variable to dimensions 1 and 2 of the MFA as insets. Quantitative variables are as in Figure 6. Functional traits are expressed as relative abundances to normalize for the total number of reads per sample.

(B) Functional trait contribution to β-diversity (SCBD), based on KEGG metabolic pathway abundances, for younger (left) and older (right) children. Shown are the top 30 traits with greatest SCBD values for each age group, where green points correspond to traits with SCBD values that are greater than the mean SCBD values of all traits (n = 8 and 6 for younger and older children, respectively).

See also Figure S8.

our data are consistent with a developmental immaturity of the gut microbiota in Bangladeshi children.

Using microscopy, we provide the first estimates of free viral particles in stunted and non-stunted children, which are similar for all children irrespective of age and health. These estimates led to VBRs ranging between 0.01 and 0.39, which are lower than those previously reported for nutrient-rich ecosystems (Parikka et al., 2017). Although our values fit within estimates for the human gut based on metagenomics (Norman et al., 2015), these low VBRs would support lysogenic replication in the gut. Consistent with findings from previous studies of disease-associated gut phage communities, we observed more reads assigned to the Caudovirales, especially the Siphoviridae family, in stunted children relative to non-stunted ones (Norman et al., 2015; Reyes et al., 2015). Interestingly, *Lactococcus* phages, which have been associated with Parkinson's disease, were also highly abundant in stunted children younger than 23 months. In contrast, reads assigned to Microviridae and crAssphage were more abundant in non-stunted children. Although both have been identified as members of the gut microbiota of healthy

individuals (Manrique et al., 2016; Shkoporov et al., 2018), their specific roles in human health remain to be determined.

In general, phages followed the same diversity patterns as their bacterial hosts, consistent with the heterogeneity correlation hypothesis (Koskella and Brockhurst, 2014; Moreno-Gallego et al., 2019). Indeed, regardless of age, non-stunted children contained high numbers of Firmicutes and Bacteroides phages in their guts, whereas stunted children had lower abundances of Bacteroides phages and higher abundances of Proteobacteria phages (especially in the older children). These convergent diversity patterns were not observed for Actinobacteria and their phages. Armed with this diversity knowledge, we then sought to determine the prevalence of the lytic and lysogenic phage replication cycles, as there is evidence for a switch from lytic to lysogenic replication in the gut with age (Manrique et al., 2017; Scanlan, 2017). Although the underlying mechanisms remain unknown, it has been suggested that the intrinsic variability in abundance and diversity of gut bacterial communities during child development would support more lytic phages and "kill the winner" dynamics; the more stable gut bacterial communities found in adults, in terms of abundance and diversity, would support lysogenic interactions following "piggyback the winner" dynamics (Manrique et al., 2017). However, we report more temperate phages in non-stunted children in both age groups, and highest in the younger children. This suggests that stunted children harbor a gut microbiota and/or specific bacterial metabolism or physiology allowing for lytic phages and kill-the-winner dynamics. This is consistent with previous studies that report a decrease in temperate phages in animal models of IBD and in patients with IBD, where gut bacterial communities are significantly disrupted and altered (Duerkop et al., 2018). In our case, whether these patterns are due to the local inflamed gut environment, the recurrent diarrheal infections, induction of lysogens, or to specific bacterial taxa that select for lytic phages remains to be determined.

We next proceeded with anaerobic *in vitro* cross-infections of bacterial and viral communities to investigate the role of phage infection in stunting. Our MFA shows a clear clustering of cross-infection conditions distinct from our controls overall and within each age group, demonstrating that these phages are still infectious and actively shaping bacterial community structure *in vitro*. We observed limited changes in bacterial community structure in our heat-killed controls. This could be due to antibacterial effects of phage components and proteins such as endolysins (Roach and Donovan, 2015), the replication of heat-resistant phages, or our incubation conditions that could have triggered prophage induction (Abedon et al., 2001; Mirzaei and Maurice, 2017). This last observation is supported by our MFA showing the association of heat-killed controls with temperate phages. Alternatively, these changes could be due to labile compounds or peptides, the activity of temperature-resistant small molecules, metabolites, or bacterial debris, which could all selectively promote the growth of a subset of bacterial taxa.

Through cross-infection experiments, we observed age-specific interactions between bacteria and phages communities: phages from children older than 23 months failed to regulate bacterial growth, as seen by increased bacterial abundances. This is further supported by limited changes in bacterial community structure after phage exposure in this age group and differences in major functional traits that contributed the most to β-diversity. In contrast, phages from the younger age group more strongly regulated gut bacterial communities. Specifically, phages from stunted children and children undergoing stunting allowed for Proteobacteria to grow, whereas phages from non-stunted children caused an increase in Firmicutes abundance. Within these younger children, the effects of phage infection on bacterial communities were strongest when bacterial communities from non-stunted children were exposed to phages from stunted children. The changes in phage community composition were due to combined changes in species abundance and turnover. In this age group, *Lactococcus* phages contributed most to the changes in bacterial community diversity of non-stunted children: they were highly abundant in the initial fecal samples of stunted children and strongly correlated with non-stunted bacteria according to our MFA. Their contribution could result from the infection of *Lactococcus* bacteria in non-stunted children, allowing for Proteobacteria (and Enterobacteria) to grow, as suggested by the strong correlation between *Lactococcus* and Enterobacteria in the MFA. There could also be a sex-specific effect of phage communities on bacterial ones, as *Lactococcus* was more prevalent in male children, who also tended to be more breastfed. Sex and breast milk have been associated with distinct gut bacterial and phage communities (Duranti et al., 2017; Human Microbiome Project, 2012; Pannaraj et al., 2018), but more data from children with different feeding practices are warranted to determine if this could also affect bacteria-phage interactions.

Bacteria-phage interactions are complex, dynamic, and can be altered by many factors. Recently, it was shown that bacterial and phage abundances can both be maintained at high densities (Bull and Gill, 2014; Bull et al., 2014; Scanlan, 2017). The reason why regulation of gut bacterial communities by phages is age-specific remains to be determined. One possible explanation could be the inability of phages to replicate significantly in older children because either they fail to establish a spreading infection or they decline to extinction after briefly propagating. Several mechanisms can affect phage replication, including host availability, defense mechanisms, and phage-phage interactions (Bull and Gill, 2014). The changes in abundance and diversity of certain bacterial taxa with age could lead to insufficient numbers of adequate hosts to sustain high levels of phage replication (Rodríguez et al., 2015), thereby lowering the efficacy of the phage treatment in the older age group. Alternatively, the differences between these age groups could extend to distinct bacterial defense mechanisms and phage infection properties. It is possible that gut bacterial communities of children older than 23 months, benefiting from a longer exposure to their phage communities and environmental phages, have more diversified bacterial resistance mechanisms resulting from a bacteria-phage arms race coevolution (Koskella and Brockhurst, 2014; Scanlan, 2017). This more diversified range of resistance alleles in the bacterial population would limit phage infectivity (Morley et al., 2017), or allow for the emergence of a resistant bacterial subpopulation within a susceptible wild type (Bull and Gill, 2014; Koskella and Brockhurst, 2014). It is also possible that the phages in the gut of younger children are more infectious than in older children and can more effectively target their bacterial hosts (Bull and Gill, 2014; Mirzaei and Maurice, 2017). One must also consider that our experimental setup, using bacterial

and phage communities of children from different households, could limit the effect of phages. Indeed, selecting for gut phage communities from non-stunted family members, with higher similarities between bacterial and phage populations, could have increased the efficacy and outcomes of our cross-infections.

Finally, we sought to determine the relative contribution of the children's metadata (diet, age, sex, health, and stunting metrics [HAZ, WAZ, and WHZ]) on the changes in bacteria-phage interactions during the cross-infections. Diet, sex, HAZ, WAZ, and WHZ all contributed strongly to shaping bacteria-phage interactions. The effects of these factors on gut bacterial communities have been reported numerous times, and their effect on phage communities might result from the changes in bacterial communities (Human Microbiome Project, 2012; Turnbaugh et al., 2009; Vonaesch et al., 2018). Yet, the significant contribution of the Gokushovirinae to bacteria-phage interactions could suggest a direct effect of these variables on phage communities, which remains to be further tested. We found the effects of these factors to be more important for children older than 23 months, most probably due to the weaning process and diversity of solid diets. Our analyses also show distinct differences between the relative contributions of variables associated with younger children from those associated with older ones.

With this study, we determined on a community level that gut phages are distinct in non-stunted and stunted children, and that phage communities isolated from unrelated children can alter the abundance and diversity of gut bacterial communities in an age-specific manner *in vitro*. Importantly, phages from stunted children allowed for Proteobacteria to grow in gut bacterial communities from non-stunted children, hinting at a possible role for phages in the pathophysiology of child stunting. Our pooled sequencing approach precludes the identification of specific bacteria-phage interactions on an individual level but shows community-level interactions that should be further pursued. We observed more active bacteria-phage interactions in early child development, suggesting an intervention time window for microbiome manipulation under the age of 23 months. Going forward, it will be necessary to validate our results *in vivo*, as our data show a strong contribution of host associated factors. It is thus highly likely that other aspects of human health, such as the host immune response and gut inflammation, will also affect bacteria-phage interactions. Using mouse models of stunting (Brown et al., 2015), one could determine if gut phage communities from stunted children enhance the stunting phenotype or pro-inflammatory gut conditions. It will also be crucial to better assess age-specific effects, to refine a possible intervention window, as well as the specific bacteria-phage interactions involved.

STAR★METHODS

Detailed methods are provided in the online version of this paper and include the following:

- KEY RESOURCES TABLE
- LEAD CONTACT AND MATERIAL AVAILABILITY
- EXPERIMENTAL MODEL AND SUBJECT DETAILS
  - Study Site and Volunteer Recruitment
- METHOD DETAILS
  - Sample Collection and Storage
  - Isolation of Phages and Bacteria from Stool
  - Bacterial and Phage Abundances
  - *In Vitro* Cross Infections
  - DNA Extraction and Sequencing
  - Metagenome Assembly and Analysis
  - Functional Profiles
  - 16S rRNA Gene Amplicon Identification
- QUANTIFICATION AND STATISTICAL ANALYSIS
- DATA AND CODE AVAILABILITY

SUPPLEMENTAL INFORMATION

Supplemental Information can be found online at https://doi.org/10.1016/j.chom.2020.01.004.

ACKNOWLEDGMENTS

This work was funded by the Bill and Melinda Gates Foundation (OPP1139814) and the Canada Research Chair Program (950-230748 X-242502) to C.F.M. J.R. and L.D. are funded by the EU ERC StG (803077/PHARMS). The authors thank members of the Maurice lab, J. Shapiro, E. Haggård-ljungquist, H.J. Haiser, and H. Foroughi-Asl for their constructive comments. The graphical abstract was made using Smart Medical Art (SMART): https://smart.servier.com.

AUTHOR CONTRIBUTIONS

M.K.M., D.M., and C.F.M. designed the research; M.K.M., M.A.A.K., P.G., and R.C. performed the experiments; M.K.M., M.T., Z.E.T., M.A.A.K., J.R., L.D., and C.F.M. analyzed the data; M.M.K. oversaw volunteer recruitment and sample coordination; M.K.M., Z.E.T., and C.F.M. wrote the paper. All authors approved this version of the manuscript.

DECLARATION OF INTERESTS

The authors declare no competing interests.

Received: June 6, 2019
Revised: October 2, 2019
Accepted: January 9, 2020
Published: February 12, 2020

REFERENCES

Abedon, S.T., Herschler, T.D., and Stopar, D. (2001). Bacteriophage latent-period evolution as a response to resource availability. Appl. Environ. Microbiol. *67*, 4233–4241.

Ahmed, T., Mahfuz, M., Islam, M.M., Mondal, D., Hossain, M.I., Ahmed, A.S., Tofail, F., Gaffar, S.A., Haque, R., Guerrant, R.L., and Petri, W.A. (2014). The MAL-ED cohort study in Mirpur, Bangladesh. Clin. Infect. Dis. *59*, S280–S286.

Bhowmik, K.R., and Das, S. (2017). On exploring and ranking risk factors of child malnutrition in Bangladesh using multiple classification analysis. BMC Nutr *3*, 73.

Blanton, L.V., Charbonneau, M.R., Salih, T., Barratt, M.J., Venkatesh, S., Ilkaveya, O., Subramanian, S., Manary, M.J., Trehan, I., Jorgensen, J.M., et al. (2016). Gut bacteria that prevent growth impairments transmitted by microbiota from malnourished children. Science *351*, aad3311.

Borcard, D., Gillet, F., and Legendre, P. (2018). Numerical Ecology with R (Use R!), Second Edition (Springer), pp. 1–300.

Brown, E.M., Wlodarska, M., Willing, B.P., Vonaesch, P., Han, J., Reynolds, L.A., Arrieta, M.C., Uhrig, M., Scholz, R., Partida, O., et al. (2015). Diet and specific microbial exposure trigger features of environmental enteropathy in a novel murine model. Nat. Commun *6*, 7806.

Buchfink, B., Xie, C., and Huson, D.H. (2015). Fast and sensitive protein alignment using DIAMOND. Nat. Methods *12*, 59–60.

Budge, S., Parker, A.H., Hutchings, P.T., and Garbutt, C. (2019). Environmental enteric dysfunction and child stunting. Nutr. Rev. *77*, 240–253.

Bull, J.J., and Gill, J.J. (2014). The habits of highly effective phages: population dynamics as a framework for identifying therapeutic phages. Front. Microbiol. *5*, 618.

Bull, J.J., Vegge, C.S., Schmerer, M., Chaudhry, W.N., and Levin, B.R. (2014). Phenotypic resistance and the dynamics of bacterial escape from phage control. PLoS One *9*, e94690.

Caporaso, J.G., Kuczynski, J., Stombaugh, J., Bittinger, K., Bushman, F.D., Costello, E.K., Fierer, N., Peña, A.G., Goodrich, J.K., Gordon, J.I., et al. (2010). QIIME allows analysis of high-throughput community sequencing data. Nat. Methods *7*, 335–336.

Chevreux, B., Pfisterer, T., Drescher, B., Driesel, A.J., Müller, W.E., Wetter, T., and Suhai, S. (2004). Using the miraEST assembler for reliable and automated mRNA transcript assembly and SNP detection in sequenced ESTs. Genome Res. *14*, 1147–1159.

Darling, A.C., Mau, B., Blattner, F.R., and Perna, N.T. (2004). Mauve: multiple alignment of conserved genomic sequence with rearrangements. Genome Res. *14*, 1394–1403.

Delcher, A.L., Bratke, K.A., Powers, E.C., and Salzberg, S.L. (2007). Identifying bacterial genes and endosymbiont DNA with Glimmer. Bioinformatics *23*, 673–679.

Dinh, D.M., Ramadass, B., Kattula, D., Sarkar, R., Braunstein, P., Tai, A., Wanke, C.A., Hassoun, S., Kane, A.V., Naumova, E.N., et al. (2016). Longitudinal analysis of the intestinal microbiota in persistently stunted young children in South India. PLoS One *11*, e0155405.

Duerkop, B.A., Kleiner, M., Paez-Espino, D., Zhu, W., Bushnell, B., Hassell, B., Winter, S.E., Kyrpides, N.C., and Hooper, L.V. (2018). Murine colitis reveals a disease-associated bacteriophage community. Nat. Microbiol. *3*, 1023–1031.

Duranti, S., Lugli, G.A., Mancabelli, L., Armanini, F., Turroni, F., James, K., Ferretti, P., Gorfer, V., Ferrario, C., Milani, C., et al. (2017). Maternal inheritance of bifidobacterial communities and bifidophages in infants through vertical transmission. Microbiome *5*, 66.

Džunková, M., Low, S.J., Daly, J.N., Deng, L., Rinke, C., and Hugenholtz, P. (2019). Defining the human gut host-phage network through single-cell viral tagging. Nat. Microbiol. *4*, 2192–2203.

Edgar, R.C., Haas, B.J., Clemente, J.C., Quince, C., and Knight, R. (2011). UCHIME improves sensitivity and speed of chimera detection. Bioinformatics *27*, 2194–2200.

Harper, K.M., Mutasa, M., Prendergast, A.J., Humphrey, J., and Manges, A.R. (2018). Environmental enteric dysfunction pathways and child stunting: a systematic review. PLoS Negl. Trop. Dis. *12*, e0006205.

Human Microbiome Project Consortium (2012). Structure, function and diversity of the healthy human microbiome. Nature *486*, 207–214.

Juarez, G.E., and Galván, E.M. (2018). Role of nutrient limitation in the competition between uropathogenic strains of Klebsiella pneumoniae and Escherichia coli in mixed biofilms. Biofouling *34*, 287–298.

Koenig, J.E., Spor, A., Scalfone, N., Fricker, A.D., Stombaugh, J., Knight, R., Angenent, L.T., and Ley, R.E. (2011). Succession of microbial consortia in the developing infant gut microbiome. Proc. Natl. Acad. Sci. USA *108*, 4578–4585.

Kortright, K.E., Chan, B.K., Koff, J.L., and Turner, P.E. (2019). Phage therapy: a renewed approach to combat antibiotic-resistant bacteria. Cell Host Microbe *25*, 219–232.

Koskella, B., and Brockhurst, M.A. (2014). Bacteria-phage coevolution as a driver of ecological and evolutionary processes in microbial communities. FEMS Microbiol. Rev. *38*, 916–931.

Krajmalnik-Brown, R., Ilhan, Z.E., Kang, D.W., and DiBaise, J.K. (2012). Effects of gut microbes on nutrient absorption and energy regulation. Nutr. Clin. Pract *27*, 201–214.

Langmead, B., and Salzberg, S.L. (2012). Fast gapped-read alignment with Bowtie 2. Nat. Methods *9*, 357–359.

Li, J., Jia, H., Cai, X., Zhong, H., Feng, Q., Sunagawa, S., Arumugam, M., Kultima, J.R., Prifti, E., Nielsen, T., et al. (2014). An integrated catalog of reference genes in the human gut microbiome. Nat. Biotechnol *32*, 834–841.

Li, L., Kapoor, A., Slikas, B., Bamidele, O.S., Wang, C., Shaukat, S., Masroor, M.A., Wilson, M.L., Ndjango, J.B., Peeters, M., et al. (2010). Multiple diverse circoviruses infect farm animals and are commonly found in human and chimpanzee feces. J. Virol *84*, 1674–1682.

Lundeen, E.A., Stein, A.D., Adair, L.S., Behrman, J.R., Bhargava, S.K., Dearden, K.A., Gigante, D., Norris, S.A., Richter, L.M., Fall, C.H., et al. (2014). Height-for-age z scores increase despite increasing height deficits among children in 5 developing countries. Am. J. Clin. Nutr *100*, 821–825.

Ma, Y., You, X., Mai, G., Tokuyasu, T., and Liu, C. (2018). A human gut phage catalog correlates the gut phageome with type 2 diabetes. Microbiome *6*, 24.

Magoč, T., and Salzberg, S.L. (2011). FLASH: fast length adjustment of short reads to improve genome assemblies. Bioinformatics *27*, 2957–2963.

MAL-ED Network Investigators.. (2017). Relationship between growth and illness, enteropathogens and dietary intakes in the first 2 years of life: findings from the MAL-ED birth cohort study. BMJ Glob. Health *2*, e000370.

Manrique, P., Bolduc, B., Walk, S.T., van der Oost, J., de Vos, W.M., and Young, M.J. (2016). Healthy human gut phageome. Proc. Natl. Acad. Sci. USA *113*, 10400–10405.

Manrique, P., Dills, M., and Young, M.J. (2017). The human gut phage community and its implications for health and disease. Viruses *9*, E141.

Maurice, C.F., Haiser, H.J., and Turnbaugh, P.J. (2013). Xenobiotics shape the physiology and gene expression of the active human gut microbiome. Cell *152*, 39–50.

Menon, P., Bamezai, A., Subandoro, A., Ayoya, M.A., and Aguayo, V.M. (2015). Age-appropriate infant and young child feeding practices are associated with child nutrition in India: insights from nationally representative data. Matern. Child Nutr *11*, 73–87.

Milani, C., Duranti, S., Bottacini, F., Casey, E., Turroni, F., Mahony, J., Belzer, C., Delgado Palacio, S., Arboleya Montes, S., Mancabelli, L., et al. (2017). The first microbial colonizers of the human gut: composition, activities, and health implications of the infant gut microbiota. Microbiol. Mol. Biol. Rev. *81*, e00036–17.

Million, M., Diallo, A., and Raoult, D. (2017). Gut microbiota and malnutrition. Microb. Pathog *106*, 127–138.

Minot, S., Bryson, A., Chehoud, C., Wu, G.D., Lewis, J.D., and Bushman, F.D. (2013). Rapid evolution of the human gut virome. Proc. Natl. Acad. Sci. USA *110*, 12450–12455.

Minot, S., Sinha, R., Chen, J., Li, H., Keilbaugh, S.A., Wu, G.D., Lewis, J.D., and Bushman, F.D. (2011). The human gut virome: inter-individual variation and dynamic response to diet. Genome Res. *21*, 1616–1625.

Mirzaei, M.K., and Maurice, C.F. (2017). Ménage à trois in the human gut: interactions between host, bacteria and phages. Nat. Rev. Microbiol. *15*, 397–408.

Monira, S., Nakamura, S., Gotoh, K., Izutsu, K., Watanabe, H., Alam, N.H., Endtz, H.P., Cravioto, A., Ali, S.I., Nakaya, T., et al. (2011). Gut microbiota of healthy and malnourished children in Bangladesh. Front. Microbiol. *2*, 228.

Moreno-Gallego, J.L., Chou, S.P., Di Rienzi, S.C., Goodrich, J.K., Spector, T.D., Bell, J.T., Youngblut, N.D., Hewson, I., Reyes, A., and Ley, R.E. (2019). Virome diversity correlates with intestinal microbiome diversity in adult monozygotic twins. Cell Host Microbe *25*, 261–272.e5.

Morley, D., Broniewski, J.M., Westra, E.R., Buckling, A., and van Houte, S. (2017). Host diversity limits the evolution of parasite local adaptation. Mol. Ecol *26*, 1756–1763.

Müller, O., and Krawinkel, M. (2005). Malnutrition and health in developing countries. CMAJ *173*, 279–286.

Nisbett, N., Davis, P., Yosef, S., and Akhtar, N. (2017). Bangladesh's story of change in nutrition: strong improvements in basic and underlying determinants with an unfinished agenda for direct community level support. Global Food Security *13*, 21–29.

Norman, J.M., Handley, S.A., Baldridge, M.T., Droit, L., Liu, C.Y., Keller, B.C., Kambal, A., Monaco, C.L., Zhao, G., Fleshner, P., et al. (2015). Disease-specific alterations in the enteric virome in inflammatory bowel disease. Cell *160*, 447–460.

Ottman, N., Smidt, H., de Vos, W.M., and Belzer, C. (2012). The function of our microbiota: who is out there and what do they do? Front Cell Infect Microbiol. *2*, 104.

Pannaraj, P.S., Ly, M., Cerini, C., Saavedra, M., Aldrovandi, G.M., Saboory, A.A., Johnson, K.M., and Pride, D.T. (2018). Shared and distinct features of human milk and infant stool viromes. Front. Microbiol. *9*, 1162.

Parikka, K.J., Le Romancer, M., Wauters, N., and Jacquet, S. (2017). Deciphering the virus-to-prokaryote ratio (VPR): insights into virus-host relationships in a variety of ecosystems. Biol. Rev. Camb. Philos. Soc. *92*, 1081–1100.

Reyes, A., Blanton, L.V., Cao, S., Zhao, G., Manary, M., Trehan, I., Smith, M.I., Wang, D., Virgin, H.W., Rohwer, F., and Gordon, J.I. (2015). Gut DNA viromes of Malawian twins discordant for severe acute malnutrition. Proc. Natl. Acad. Sci. USA *112*, 11941–11946.

Reyes, A., Wu, M., McNulty, N.P., Rohwer, F.L., and Gordon, J.I. (2013). Gnotobiotic mouse model of phage-bacterial host dynamics in the human gut. Proc. Natl. Acad. Sci. USA *110*, 20236–20241.

Roach, D.R., and Donovan, D.M. (2015). Antimicrobial bacteriophage-derived proteins and therapeutic applications. Bacteriophage *5*, e1062590.

Robertson, R.C., Manges, A.R., Finlay, B.B., and Prendergast, A.J. (2019). The human microbiome and child growth - first 1000 days and beyond. Trends Microbiol. *27*, 131–147.

Rodríguez, J.M., Murphy, K., Stanton, C., Ross, R.P., Kober, O.I., Juge, N., Avershina, E., Rudi, K., Narbad, A., Jenmalm, M.C., et al. (2015). The composition of the gut microbiota throughout life, with an emphasis on early life. Microb. Ecol. Health Dis. *26*, 26050.

Roux, S., Krupovic, M., Poulet, A., Debroas, D., and Enault, F. (2012). Evolution and diversity of the Microviridae viral family through a collection of 81 new complete genomes assembled from virome reads. PLoS One *7*, e40418.

Sanin, K.I., Islam, M.M., Mahfuz, M., Ahmed, A.M.S., Mondal, D., Haque, R., and Ahmed, T. (2018). Micronutrient adequacy is poor, but not associated with stunting between 12-24 months of age: a cohort study findings from a slum area of Bangladesh. PLoS One *13*, e0195072.

Scanlan, P.D. (2017). Bacteria-bacteriophage coevolution in the human gut: implications for microbial diversity and functionality. Trends Microbiol. *25*, 614–623.

Shkoporov, A.N., and Hill, C. (2019). Bacteriophages of the human gut: the "known unknown" of the microbiome. Cell Host Microbe *25*, 195–209.

Shkoporov, A.N., Khokhlova, E.V., Fitzgerald, C.B., Stockdale, S.R., Draper, L.A., Ross, R.P., and Hill, C. (2018). ΦCrAss001 represents the most abundant bacteriophage family in the human gut and infects Bacteroides intestinalis. Nat. Commun *9*, 4781.

Subramanian, S., Huq, S., Yatsunenko, T., Haque, R., Mahfuz, M., Alam, M.A., Benezra, A., DeStefano, J., Meier, M.F., Muegge, B.D., et al. (2014). Persistent gut microbiota immaturity in malnourished Bangladeshi children. Nature *510*, 417–421.

Sutton, T.D.S., Clooney, A.G., Ryan, F.J., Ross, R.P., and Hill, C. (2019). Choice of assembly software has a critical impact on virome characterisation. Microbiome *7*, 12.

Tetz, G., Brown, S.M., Hao, Y., and Tetz, V. (2018). Parkinson's disease and bacteriophages as its overlooked contributors. Sci. Rep *8*, 10812.

Thompson, L.R., Zeng, Q., Kelly, L., Huang, K.H., Singer, A.U., Stubbe, J., and Chisholm, S.W. (2011). Phage auxiliary metabolic genes and the redirection of cyanobacterial host carbon metabolism. Proc. Natl. Acad. Sci. USA *108*, E757–E764.

Turnbaugh, P.J., Ridaura, V.K., Faith, J.J., Rey, F.E., Knight, R., and Gordon, J.I. (2009). The effect of diet on the human gut microbiome: a metagenomic analysis in humanized gnotobiotic mice. Sci. Transl. Med *1*, 6ra14.

Van Belleghem, J.D., Dąbrowska, K., Vaneechoutte, M., Barr, J.J., and Bollyky, P.L. (2018). Interactions between bacteriophage, bacteria, and the mammalian immune system. Viruses *11*, E10.

Vonaesch, P., Morien, E., Andrianonimiadana, L., Sanke, H., Mbecko, J.R., Huus, K.E., Naharimanananirina, T., Gondje, B.P., Nigatoloum, S.N., Vondo, S.S., et al. (2018). Stunted childhood growth is associated with decompartmentalization of the gastrointestinal tract and overgrowth of oropharyngeal taxa. Proc. Natl. Acad. Sci. USA *115*, E8489–E8498.

Yutin, N., Makarova, K.S., Gussow, A.B., Krupovic, M., Segall, A., Edwards, R.A., and Koonin, E.V. (2018). Discovery of an expansive bacteriophage family that includes the most abundant viruses from the human gut. Nat. Microbiol. *3*, 38–46.

STAR★METHODS

KEY RESOURCES TABLE

REAGENT or RESOURCE	SOURCE	IDENTIFIER
Biological Samples		
Bangladeshi infant stool samples	This study	N/A
Chemicals, Peptides, and Recombinant Proteins		
0.02 um filter, Anodisc	Whatman, GE Healthcare	Cat No./6809-6002
0.22 um syringe filter	Merck-Millipore, USA	Cat No./SLGP033RK
0.4 um filter, Cyclopore	Whatman, GE Healthcare	Cat No./7060-2504
AnaeroGen Gas pack, Oxoid AN0025A	Thermo Scientific	Cat No./AN0025A
DNase I	Invitrogen, Life Technologies	Cat No./AM2222
Lysozyme (100 mg/mL)	Thermo Scientific	Cat No./90082
Oxygen indicator dry strip	BD	Cat No./271055
Protease K (20 mg/ml)	Thermo Scientific	Cat No./AM2548
SybrGold	Invitrogen, Life Technologies	Cat No./S11494
Critical Commercial Assays		
DNeasy Powersoil Kit	Qiagen	Cat No./ID: 12888-100
Genomic DNA Clean & Concentrator	ZYMO research	Cat No./ID: D4064
illustra Ready-To-Go Genomiphi Kit	GE healthcare	Cat No./ID: 25-6601-96
MiSeq PE250 instrument	Genome Quebec	http://gqinnovationcenter.com/index.aspx
Deposited Data		
Raw and analyzed data for bacterial and phage metagenomes	This paper	SRA: PRJNA594824
Raw and analyzed data for bacterial 16S (V4 region)	This paper	SRA: PRJNA595506
Oligonucleotides		
16S rDNA barcodes and primers	Table S3	Table S3
Software and Algorithms		
Bowtie2	Langmead and Salzberg, 2012	http://bowtie-bio.sourceforge.net/bowtie2/index.shtml
BBMerge	N/A	https://jgi.doe.gov/data-and-tools/bbtools/
DIAMOND v.0.9.21.122	Buchfink et al., 2015	https://ab.inf.uni-tuebingen.de/software/diamond/
Geneious 11	N/A	https://www.geneious.com/
Glimmer	Delcher et al., 2007	https://ccb.jhu.edu/software/glimmer/
Mauve	Darling et al., 2004	http://darlinglab.org/mauve/mauve.html
MIRA	Chevreux et al., 2004	https://sourceforge.net/projects/mira-assembler/
Python programming language	Python Software Foundation	https://www.python.org
Python library Matplotlib	N/A	https://matplotlib.org
Python library Numpy v1.12.1	N/A	http://www.numpy.org/
Python library Pandas v.0.20.1	N/A	https://pandas.pydata.org/
R Statistical Computing Software	The R Foundation	https://www.r-project.org/
adespatial R package	N/A	https://cran.r-project.org/web/packages/adespatial/index.html
FactoMine R package	N/A	http://factominer.free.fr
stats R package	N/A	https://stat.ethz.ch/R-manual/R-devel/library/stats/html/00Index.html
vegan R package	N/A	https://cran.r-project.org/web/packages/vegan/index.html

(*Continued on next page*)

Continued

REAGENT or RESOURCE	SOURCE	IDENTIFIER
QIIME2	Caporaso et al., 2010	https://docs.qiime2.org/2019.1/
UCHIME	Edgar et al., 2011	https://drive5.com/usearch/manual/uchime_algo.html

LEAD CONTACT AND MATERIAL AVAILABILITY

Further information and requests for resources and reagents should be directed to and will be fulfilled by the Lead Contact, Corinne Maurice (corinne.maurice@mcgill.ca). This study did not generate new unique reagents.

EXPERIMENTAL MODEL AND SUBJECT DETAILS

Study Site and Volunteer Recruitment

A slum area in Mirpur (location: 23° 48′ 33.51″ N, 90° 21′ 39.34″ E) located in the capital city of Dhaka, Bangladesh was selected as the study site, as detailed elsewhere (Ahmed et al., 2014). Because diet is a major regulator of the gut microbiota and is tightly linked to child growth, we explored the bacteriaphage dynamics in two groups of children differing in their age-specific diet and having either stunted or normal growth. Anthropometric measurements (height, weight) were taken following a cross-sectional prescreening of children aged between 6-23 months and 24-48 months. These age groups were preliminary targeted because of the strong dietary differences between them. However, upon observation of feeding behavior and recruitment of children at the study site during cross-sectional screening, children enrolled in the younger age group were between 14-23 months old, age-sex matched, and fed a mixture of milk and solid food. Children in the older age group had entirely transitioned to solid food for more than 2 months, were between 28-38 months old, and had a boy-girl ratio of 2:3. Sampling children of both sexes also allowed us to determine if sex was associated with any of our observations. Each group consisted of 15 stunted or undergoing stunting children, and 15 non-stunted children. Nutritional status related to height-for-age Z (HAZ), weight-for-age Z (WAZ) and weight-for-height Z (WHZ) scores were assessed using the WHO growth reference for the same age and sex (WHO Anthro software, version 3.2.2, WHO, Geneva, Switzerland). The WHO-recommended HAZ cut-off point of <-2 SD for low height-for-age status was used to define stunting (i.e., when HAZ is below -2 SD of the reference value) in this study (Bhowmik and Das, 2017; Sanin et al., 2018). Children selected for this study had not taken any nutritional supplements, did not receive any antibiotics treatment, nor had a diarrheal episode at least one month prior to sampling. Fecal samples from a total of 60 children (30 stunted and 30 non-stunted) were collected for the study in a pair-wise manner (equal number of stunted and non-stunted samples at a time per group). Sampling was performed between November 2016 and August 2017. This study was conducted following the IRB approved studies: A04M2715B (McGill University) and PR-16001 (icddr,b). Because of the young age of the participants, consent was obtained from their parents/legal guardian, who were provided with oral and written information about the project and research in English and in Bengali. Participation was entirely voluntary.

METHOD DETAILS

Sample Collection and Storage

Immediately after sample collection (<5 min), a portion of the fresh stool sample was placed inside a 50mL Falcon tube with a gas exchange lid (Bio-reaction tube, Ultident cat# 229475), which was then rapidly placed inside an anoxic Ziplock bag containing an AnaeroGen Gas pack (Oxoid AN0025A), typically used for the storage of clinical oxygen-sensitive samples. To limit oxygen exposure, a new gas pack was placed inside and the bag was sealed (<1 min). The absence of oxygen was visually confirmed with an oxygen indicator dry strip (BD BBL). Within 3 hours of sample collection, samples were brought back to the icddr,b, where they were immediately placed in a portable glove box with nitrogen and mixed gas (5% H_2, 20% CO_2, balance nitrogen). All subsequent sample-processing steps were performed anaerobically in pre-reduced buffers and media containing cysteine.

Isolation of Phages and Bacteria from Stool

Samples from each donor, non-stunted and stunted, were mixed 1 to 10 W:V in reduced Phosphate-buffered saline (rPBS), and vortexed thoroughly to separate phages and bacterial communities from large organic particles matter in the samples. The achieved suspensions were subsequently centrifuged at 700 g for 1 minute, to remove larger organic particles, followed by a 30-minute centrifugation at 3,200g to pellet the bacterial community and separate them from phages. Phage supernatants were transferred to a new tube and bacterial pellets were resuspended in reduced 10% BHI. The phage supernatants were passed through sterile syringe filters (0.22 um, Millex-GP, Merck-Millipore, USA) to separate phages from bacteria that remained in the supernatant. One aliquot of the phage samples from both health status was heat-killed by incubation at 95°C for 20min and 10 min at -80°C followed by DNase (DNase I, Invitrogen, Life Technologies) treatment for 2 h, as a control for the effect of the live phages in the experiment.

Bacterial and Phage Abundances

Samples from the same age group were pooled randomly for phage and bacterial enumeration via epifluorescence microscopy (pool size was 3-5 samples). Briefly, phage and bacterial samples were fixed by 1% formaldehyde and diluted in Tris-EDTA buffer. Diluted samples were filtered on 0.4 um (Cyclopore®, Whatman, GE Healthcare) or 0.02 um (Anodisc™, Whatman, GE Healthcare) filters and stained with SybrGold (2.5X final concentration, Invitrogen, Life Technologies) for bacterial and phage enumeration, respectively. Bacterial and phage filters were made in triplicate, except for samples from the younger children, which were done in five replicates. An optical fluorescence microscope (Olympus BX53, Japan) was used to analyze the phage and bacterial filters, and 20 fields of view on average were counted. Bacterial and phage counts were performed on the same day to ensure correct sample dilution for the *in vitro* cross infection experiments.

In Vitro Cross Infections

A total of 15 experiments were performed on each age group (younger age group: 14-23 months, older age group: 28-38 months). Each of the *in vitro* cross infection experiments was individually performed under anaerobic conditions. On every sampling day, phages and bacterial communities were isolated from fresh fecal samples of one non-stunted and one stunted child via consecutive centrifugation and filtration. The isolated bacterial communities were diluted (~100x in rPBS) to reach the concentration of ~10e8.ml^{-1}. A similar dilution factor was applied to the phages to ensure bacterial and phage communities were mixed in abundances similar to those determined in the initial stool samples. We proceeded with a full factorial design, where the gut bacterial community from a non-stunted donor was cross-infected with phages from a stunted donor, and vice versa, for 12 hours at 37°C in a diluted nutrient-rich reduced media (10% BHI, brain heart infusion broth) to limit the effect of *in vitro* growth on bacteria-phage interactions. Controls for each cross-infection experiment consisted of (1) non-stunted bacteria with non-stunted phages, (2) stunted bacteria with stunted phages, as well as (3) non-stunted bacteria with heat-killed stunted phages, and (4) stunted bacteria with heat-killed non-stunted phages. Heat-killing effectively reduced the number of phages (free VLPs), which were undetectable at the start of the cross-infection experiments (Figures 4A and 4B).

DNA Extraction and Sequencing

To acquire community-level information and obtain enough material for bacterial and phage metagenomics sequencing, fecal samples were pooled as follows: all the original samples from each age group and for each health status were pooled to generate four pools of samples for phage metagenomics and four pools for bacterial metagenomics. For 16S rDNA sequencing, we created 3 pools of 5 samples per age group per health status, leading to a total of 12 pools. The same strategy was applied to the samples after the *in vitro* cross-infections. We did not find any evidence of the effect of pooling on the bacterial community (phyla and family level) when testing for differences among our three pools with the cross-infection data (family and phyla bacteria), and controlling for sex in the Mixed Regression Tree.

Phages. Fecal samples were first mixed with PBS 1:10 w/v and thoroughly vortexed to separate phage and bacterial communities from organic biological matter. This fecal slurry was then centrifuged at 700xg for 1 minute to remove the larger particles, followed by 30 minutes of 6,000xg centrifugation to separate phages and bacteria. After cross-infections, samples were pooled across treatments and across controls to obtain sufficient genomic material for sequencing. The supernatants were subsequently passed through 0.22 um syringe filters to remove remaining bacteria. The filtrates were centrifuged at 35,000 g for 2 hours to collect the phage particles in the pellets. The phage pellets were resuspended in 1 ml of SM buffer (NaCl/MgSO4•7H2O/Tris-Cl/H2O). The supernatants were treated with 40 μL of lysozyme (50 mg.mL^{-1}) for 30 min at 37° C; followed by a DNase I treatment for 2h at 37 °C to remove non-phage derived DNA. DNase I was inactivated by heating the suspension at 65° C for 30 minutes, and left at room temperature to cool before the next enzymatic reaction. Ten ul of 20% SDS and 40 ul of protease K (20 mg.mL^{-1}) were added to each 500ul of sample, and incubated for 1h at 37° C. Subsequently, 35 ul of 5M NaCl and 28 ul of 10% CTAB/0.7 M NaCl were added to the mixture and incubated at 65 °C for 30 min. The aqueous phase was transferred to a new tube and 100% cold ethanol was added up to two times of the total volume and incubated overnight at -80 °C. Tubes were then centrifuged at 16,000g at 4 °C for 1h. Supernatants were removed, and DNA pellets were resuspended in 100 ul of Tris-EDTA buffer. The extracted DNA was purified using the Genomic DNA Clean & Concentrator, ZYMO research (Cat No./ID: D4064), according to the manufacturer's protocol. The concentration of the purified DNA was measured via Qubit, and amplified in triplicate using an Illustra Ready-To-Go Genomiphi Kit, GE, (Cat No./ID: 25-6601-96) according to the manufacturer's protocol. Amplification replicates were combined and the isolated phage DNA was sequenced using an Illumina MiSeq PE250 at the McGill University and Genome Quebec Innovation Center (http://gqinnovationcenter.com/index.aspx).

Bacteria. Isolated bacterial samples were pooled to make three biological replicates (three replicates per age group and health status). Bacterial DNA was extracted using DNeasy Powersoil Kit, Qiagen (Cat No./ID: 12888-100) according to the manufacturer's protocol. The v4 region of the 16S rDNA gene was amplified using triplicate PCR reactions with custom barcoded primers and the pooled amplicons were sequenced as previously detailed (Maurice et al., 2013). We also proceeded with shotgun sequencing using an Illumina MiSeq PE250.

Metagenome Assembly and Analysis

An overview of the metagenomics analysis is provided Figure S1B. On average 638,978 reads per sample were generated by sequencing for phages; and 789,495 reads per sample for bacteria. For phage analysis, we excluded reads showing nucleotide

identity to human genomes (Whole Genome Shotgun, NCBI), representing 0.5% of all reads. A small proportion (2.6%) of the remaining contigs showed nucleotide identity to the NCBI Bacterial databases. Phages are important vectors for horizontal gene transfer, and bacteria often carry one or more prophage as part of their genomes. As such, the removal of all contigs with similarity to bacterial genomes could lead to the loss of phage reads (Minot et al., 2011; Reyes et al., 2015). We therefore determined whether this small proportion of contigs also showed similarity to phage proteins. Most (86%) of these contigs showed similarity to phage proteins, and were thus included in our analyses. Raw reads produced by sequencing were trimmed for the adapters and quality checked using BBDuk (http://jgi.doe.gov/data-and-tools/bb-tools/); default options were used. Trimmed reads were subsequently merged by FLASH(Magoc and Salzberg, 2011). Dedupe (https://docs.dedupe.io/en/latest/) from BBTools was used to remove duplicate reads. We used a two-step assembly approach (step 1: De Novo assembly, step 2; Map to reference assembly) for building a cumulative contigs library. MIRA (https://sourceforge.net/projects/mira-assembler/) (Chevreux et al., 2004) was used for the De Novo assembly and Bowtie 2 (Langmead and Salzberg, 2012) and Geneious 11 (https://www.geneious.com/) were used for map to reference assembly (six steps, 3&3)(Sutton et al., 2019). UCHIME was used to remove chimera (Edgar et al., 2011). ORFs were identified for contigs using Glimmer (https://ccb.jhu.edu/software/glimmer/) (Delcher et al., 2007). All contigs longer than 1,000 bp with a minimum of one ORF were compared to a viral genome reference (downloaded on October 3, 2017, from NCBI and PHAST prophage database) using BlastX (E-value < 1e-5) for viruses, whereas Megablast was used for bacteria. When reads related to sequences of multiple taxa, a common ancestor was identified. The contigs produced for each health condition were compared using Mauve (Darling et al., 2004) and merged to make a united library to analyze the relative richness of each phage genome. To measure the relative abundance, reads were recruited to contigs generated using Geneious 11 with over 70% coverage and a minimum of 90% identity (Manrique et al., 2016; Reyes et al., 2013). The analysis was performed within Geneious 11. Temperate phages were differentiated from non-temperate based on their nucleotide identity (>70% coverage and >90% identity) to known temperate phages (assigned using BlastX, E-value <1e-5) and/or the presence of lysogeny-associated genes, such as the *integrase* gene. Reads showing >90% nucleotide identity to an integrase gene reference database (downloaded on March 7, 2019 from NCBI) with over 70% reads' coverage were classified as temperate.

E. coli. While many *E. coli* strains are naturally present in the gut, several pathogenic strains have been characterized, such as Shiga toxin-producers. Contigs that showed nucleotide identity (using a cut-off value of 10e-5, Megablast) to NCBI *E. coli* sequences were determined. The quality-controlled reads were recruited (with >70% coverage and >90% identity) to the contigs assigned to different *E. coli* strains.

Functional Profiles

An overview of the functional analysis is provided in Figure S2C. Reads were trimmed for adapters and quality checked using BBDuk (https://jgi.doe.gov/data-and-tools/bbtools/); a cutoff value of 25 Q score was used. Reads shorter than 60bp were removed. Trimmed reads were merged by BBMerge (https://jgi.doe.gov/data-and-tools/bbtools/). Reads subsequently were mapped to Integrated Gene Catalogs (IGC), an integrated catalog of reference genes in the human gut microbiome (Li et al., 2014), by BLASTX using DIAMOND v.0.9.21.122 (Buchfink et al., 2015)with maximum e-value cutoff 10e3. The best hits provided by IGC were used (Moreno-Gallego et al., 2019).

16S rRNA Gene Amplicon Identification

The V4 hypervariable region was amplified using barcoded 515/806 primer pairs. For the group of younger children, an average of 242,273.7 reads per sample was sequenced (range: 57,964 to 471,403 reads per sample). For the group of older children, an average of 334,750.75 reads per sample was sequenced (range: 269,150 to 420,998 reads per sample). Trimming, merging of paired-end reads, and quality filtering were performed using DADA2. After quality filtering, the group of younger children had an average of 36,789.2 reads per sample, ranging from 11,725 to 63,290 reads per sample with 1683 unique features. For the group of older children after quality filtering, there was an average of 52,775.8 reads per sample, ranging from 35,805 to 72,291 reads per sample with 4,106 unique features. The taxonomic analysis was performed using a pre-trained Naïve Bayes classifier trained on the Greengenes database 13_8 99% OTUs. The 16S rRNA analysis preformed using QIIME2 (https://docs.qiime2.org/2019.1/) (Caporaso et al., 2010).

QUANTIFICATION AND STATISTICAL ANALYSIS

The comparison of bacterial and virus-like particles absolute abundances between age groups and health status was carried out using Prism 7 (GraphPad software) using a one way test of variance with Holm-Sidak's multiple comparisons post-hoc test. The remaining statistical analyses were conducted in the R open source software (R core team 2018; 36). In particular, the *stats* package was used to run all ANOVAs and t-tests (using the lm() and t.test() functions), the *vegan* package was used to calculate the Shannon diversity (using the diversity() function), *adespatial* was used to obtain the species and treatment contributions to β-diversity (SCBD and LCBD, respectively; using the beta.div() function), and lastly, the *FactoMineR* package was used to run the multiple factor analysis (MFA) (using the function MFA()). Briefly, an MFA is a Principal Component Analysis (PCA) applied to the entire set of variables, but where each set is weighted by their variance in the global analysis. This multivariate correlation analysis is thus useful to examine the relationships among several different types of data or factors, and as such, allowed us to assess the contribution of each factor to changes in the gut microbiome. To restrict the number of variables used in the MFA and help its interpretation, we initially only used

the bacterial species, phages species, and functional pathways which dominated the community. This was determined by conducting individuals PCAs on each matrix, and identifying the species or trait that contributed the most to the first two axes of the PCA (using the cleanplot.pca() function in R). For both the β-diversity and MFA on bacteria and phage species data, the species matrices were Hellinger transformed (square root of the Chord transformation) to control for double-zeros (that is, to avoid two observations with 0 abundance to be interpreted as strongly similar in their absence of species). Statistical details of experiments can be found in the Results section and in the figure legends.

DATA AND CODE AVAILABILITY

Metagenomes and bacterial 16S rRNA data can be accessed on the SRA database, accession numbers PRJNA: 594824 (phage and bacterial metagenomes) and PRJNA: 595506 (16S).

Cell Host & Microbe, Volume 27

Supplemental Information

Bacteriophages Isolated from Stunted Children Can Regulate Gut Bacterial Communities in an Age-Specific Manner

Mohammadali Khan Mirzaei, Md. Anik Ashfaq Khan, Prakash Ghosh, Zofia E. Taranu, Mariia Taguer, Jinlong Ru, Rajashree Chowdhury, Md. Mamun Kabir, Li Deng, Dinesh Mondal, and Corinne F. Maurice

SUPPLEMENTAL FIGURES

Figure S1. Related to all Figures. (a) Overview of experimental procedures. Schematic diagram of the experimental setup and procedures for one age group. These procedures were repeated in a second age group, leading to a total of 60 children surveyed. (b) Metagenomics workflow, with color-coded different types of analytical steps. * A cut-off value of e-value <1e-5 was used. When contigs showed similarity to different strains within one species, a common ancestor was used. ⌘ indicates that >70% coverage and >90% identity were used.

Figure S2. Related to Figures 1 and 3. Viral and bacterial abundances are similar across health status and age. Violin plots of whole community abundances of (a) Virus-Like Particles (VLPs), (b) Bacteria, and (c) the resulting Virus-to-bacteria ratio in fecal samples from 30 non-stunted and 30 stunted children aged 14-38 months. The enumeration of phage and bacterial cells was performed using epifluorescence microscopy (see Star Methods). N= 60 children total (15/age group/health status). The width of the violin plots reflects the frequency distribution of the data, dotted lines show the median and lower and upper quartiles.

Figure S3. Related to Figure 1. Shannon diversity (a, b) and richness (c, d) indices for bacterial phyla in the original samples show opposing patterns from those observed at the species level. Each index is compared among (a, c) the age according to the children's health status, and (b, d) the HAZ score according to the children's age group. In all cases, there is an interaction between the children's age and health status on Shannon diversity and richness indexes. In particular, we see that richness increases with HAZ, whereas Shannon decreases. Pools I-III: samples from the same age group were pooled randomly for sequencing (pool size was 3-5 samples).

Figure S4. Related to Figure 5. Changes in bacterial phyla Shannon evenness and richness in cross-infected cohorts among age groups. Shannon diversity (a) and richness (b) indices for bacterial phyla. Bacterial evenness decreased with age in stunted children (a), whereas the number of phyla either increased (stunted, green tones) or remained stable (non-stunted, red tones) with age (b).

Figure S5. Related to Figure 5. **Local contribution of samples to β-phage species diversity (LCBD) after cross-infections**. Shown are the LCBD *vs.* treatment type for the younger (top) and the older (bottom) age groups, where cross-infection samples with non-stunted bacteria are shown in red and those with stunted bacteria are in green.

Figure S6. Related to Figure 6. Multiple Factor Analysis of all bacterial species, phage species, and children's metadata in younger children. Quantitative variables: milk feed, age, sex, HAZ, WAZ, and WHZ; qualitative variables: health status, treatment type, and treatment factor. Shown in the first panel is the ordination of qualitative and in the second and third panel the contribution of the quantitative variables to dimensions 1 and 2, respectively, where the dashed red line separates the variables that contribute the most (higher than average contribution of all variables) to the MFA. The Hellinger transformation of the bacteria species, phage species, and phage replication cycle data are presented here.

Figure S7. Related to Figure 6. Multiple Factor Analysis of all bacterial species, phage species, and children's metadata in older children. Quantitative variables: milk feed, age, sex, HAZ, WAZ, and WHZ; qualitative variables: health status, treatment type, and treatment factor. Shown in the first panel is the ordination of qualitative and in the second and third panel the contribution of the quantitative variables to dimensions 1 and 2, respectively, where the dashed red line separates the variables that contribute the most (higher than average contribution of all variables) to the MFA. The Hellinger transformation of the bacteria species, phage species, and phage replication cycle data are presented here.

Figure S8. Related to Figure 7. Phage communities alter functional traits of bacterial community in an age-specific manner. (a) MFA of the dominant functional traits and children metadata for both age groups. Dominant functional traits shown were selected using a PCA and the contribution circle from the cleanplot.pca() function. MFA ordination of the qualitative variables (health status, treatment type, and treatment factor). The Hellinger transformation of the bacterial, phage, and replication cycle data are presented here. (b) Relationship between the local contribution of samples to beta diversity of the functional traits (LCBD) after cross-infections for bacteria from (top panels) and phages (bottom panels).

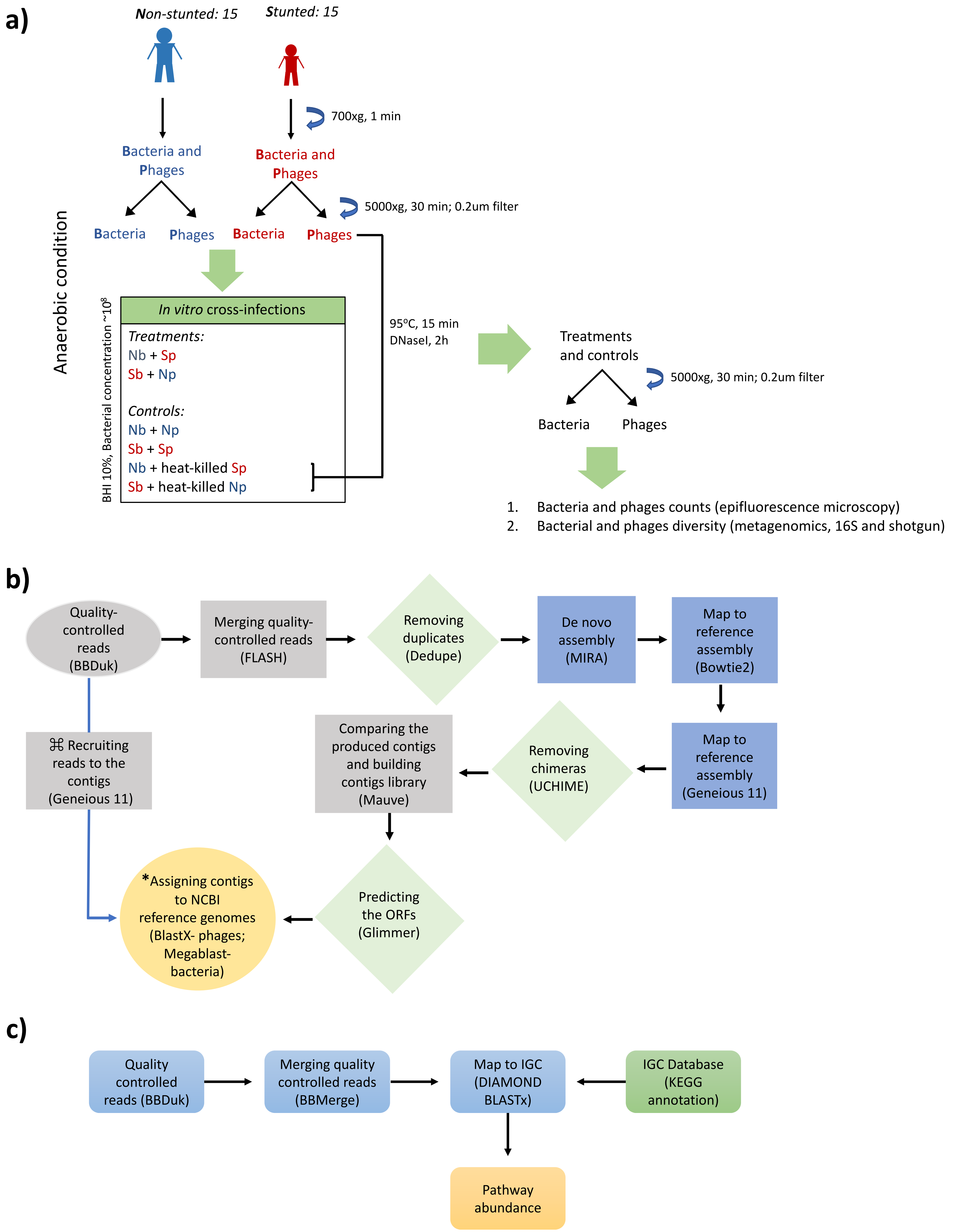

a)
Non-stunted: 15
Stunted: 15
700xg, 1 min
Bacteria and Phages
Bacteria and Phages
5000xg, 30 min; 0.2um filter
Bacteria
Phages
Bacteria
Phages
Anaerobic condition
BHI 10%, Bacterial concentration ~10^8
In vitro cross-infections
Treatments:
Nb + Sp
Sb + Np
Controls:
Nb + Np
Sb + Sp
Nb + heat-killed Sp
Sb + heat-killed Np
95°C, 15 min
DNaseI, 2h
Treatments and controls
5000xg, 30 min; 0.2um filter
Bacteria
Phages
1. Bacteria and phages counts (epifluorescence microscopy)
2. Bacterial and phages diversity (metagenomics, 16S and shotgun)
b)
Quality-controlled reads (BBDuk)
Merging quality-controlled reads (FLASH)
Removing duplicates (Dedupe)
De novo assembly (MIRA)
Map to reference assembly (Bowtie2)
Map to reference assembly (Geneious 11)
Removing chimeras (UCHIME)
Comparing the produced contigs and building contigs library (Mauve)
⌘ Recruiting reads to the contigs (Geneious 11)
Predicting the ORFs (Glimmer)
*Assigning contigs to NCBI reference genomes (BlastX- phages; Megablast- bacteria)
c)
Quality controlled reads (BBDuk)
Merging quality controlled reads (BBMerge)
Map to IGC (DIAMOND BLASTx)
IGC Database (KEGG annotation)
Pathway abundance

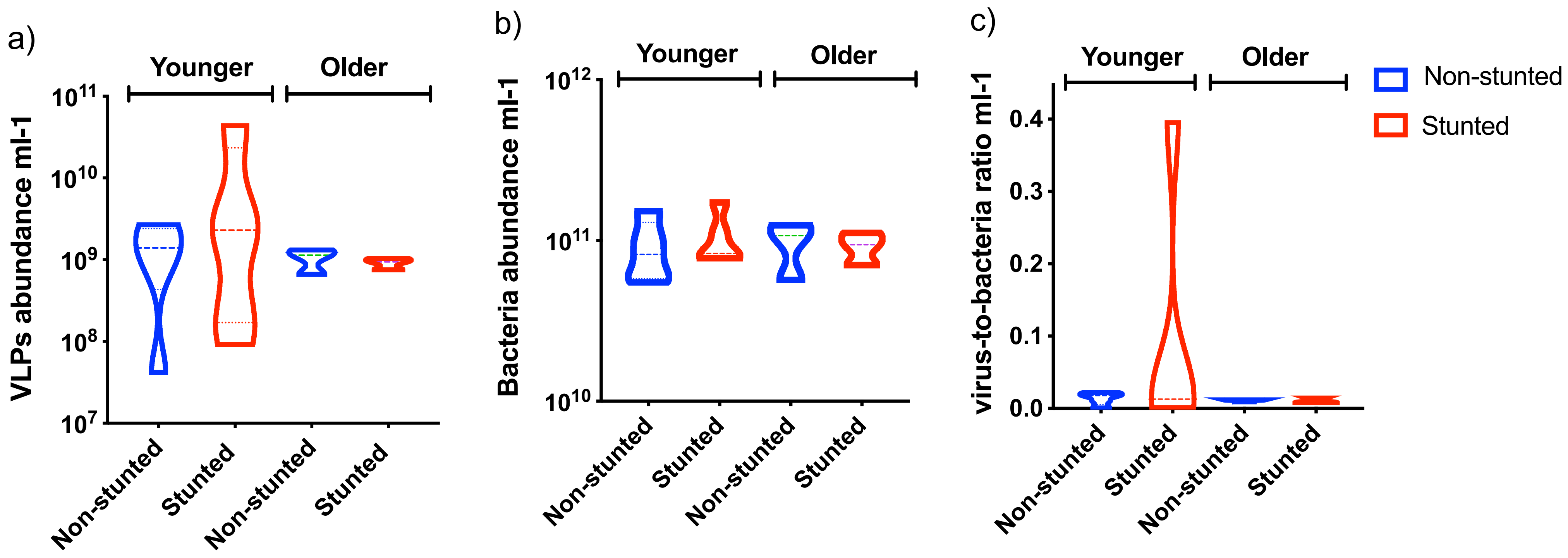

a)
Younger
Older
VLPs abundance ml-1
10^{11}
10^{10}
10^{9}
10^{8}
10^{7}
Non-stunted
Stunted
Non-stunted
Stunted
b)
Younger
Older
Bacteria abundance ml-1
10^{12}
10^{11}
10^{10}
Non-stunted
Stunted
Non-stunted
Stunted
c)
Younger
Older
virus-to-bacteria ratio ml-1
0.4
0.3
0.2
0.1
0.0
Non-stunted
Stunted
Non-stunted
Stunted
Non-stunted
Stunted

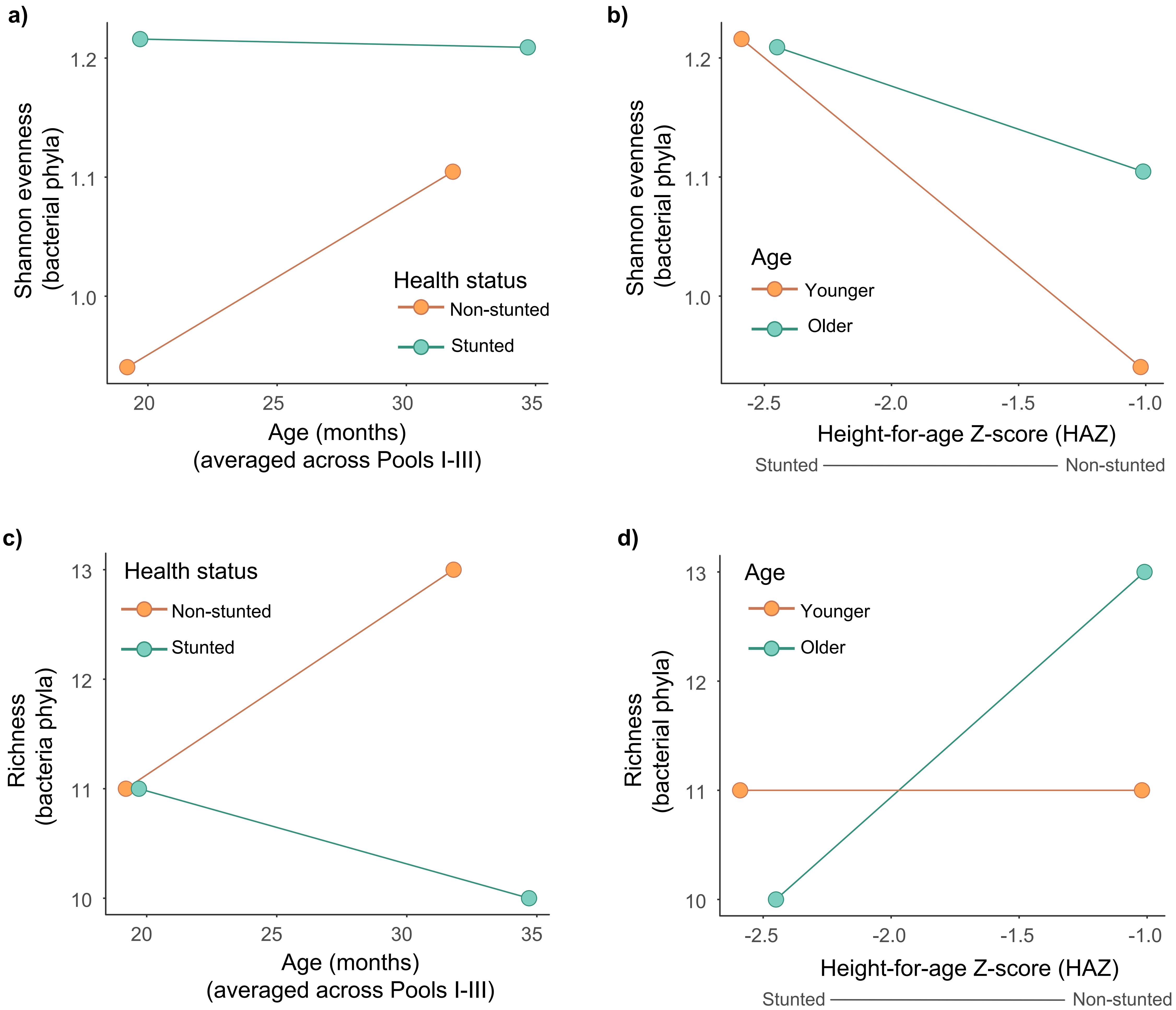

a)
Shannon evenness (bacterial phyla)
1.2
1.1
1.0
Health status
Non-stunted
Stunted
20
25
30
35
Age (months) (averaged across Pools I-III)
b)
Shannon evenness (bacterial phyla)
1.2
1.1
1.0
Age
Younger
Older
-2.5
-2.0
-1.5
-1.0
Height-for-age Z-score (HAZ)
Stunted
Non-stunted
c)
Richness (bacteria phyla)
13
12
11
10
Health status
Non-stunted
Stunted
20
25
30
35
Age (months) (averaged across Pools I-III)
d)
Richness (bacterial phyla)
13
12
11
10
Age
Younger
Older
-2.5
-2.0
-1.5
-1.0
Height-for-age Z-score (HAZ)
Stunted
Non-stunted

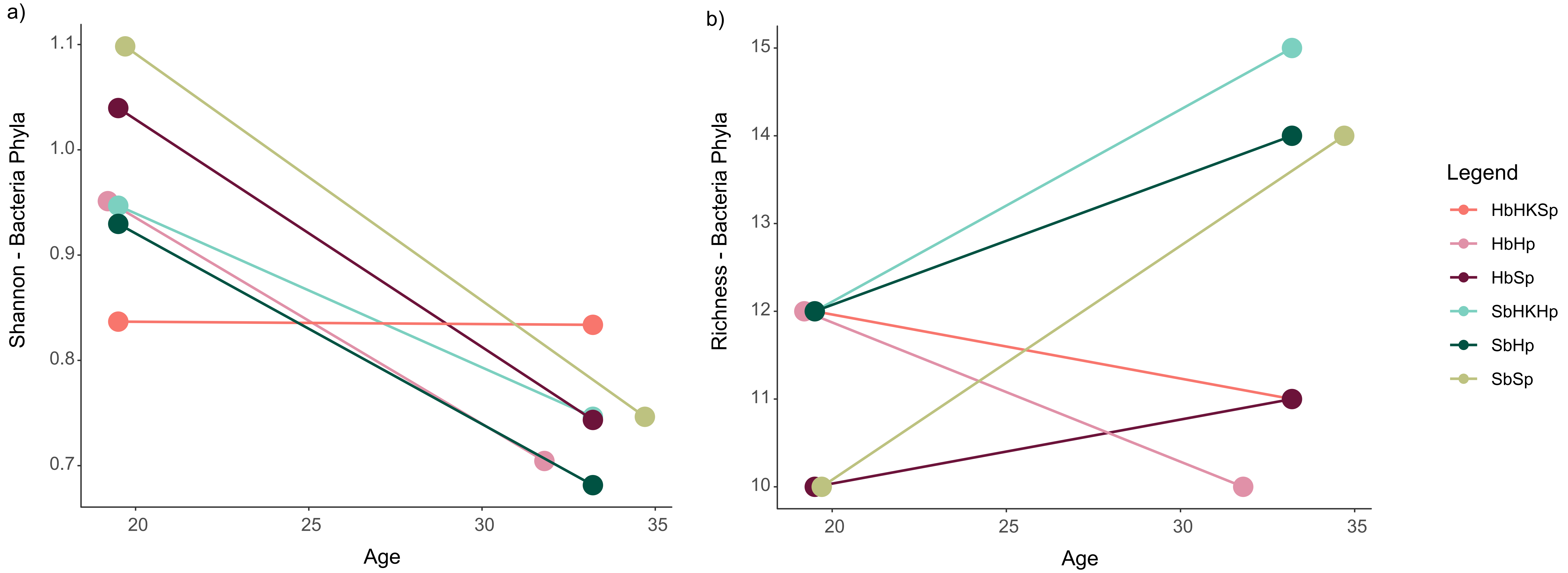

a)
Shannon - Bacteria Phyla
1.1
1.0
0.9
0.8
0.7
20
25
30
35
Age
b)
Richness - Bacteria Phyla
15
14
13
12
11
10
20
25
30
35
Age
Legend
HbHKSp
HbHp
HbSp
SbHKHp
SbHp
SbSp

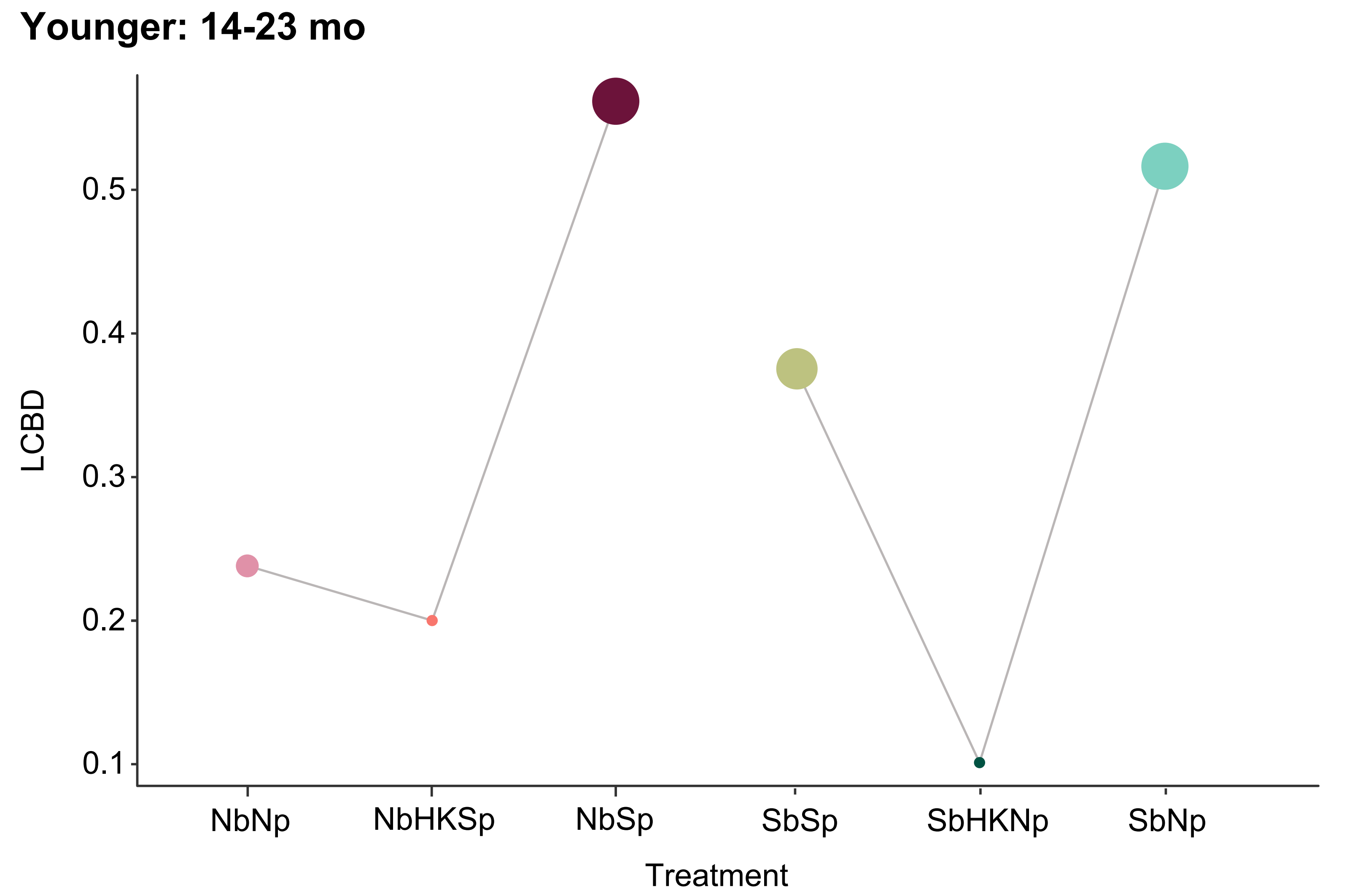
Younger: 14-23 mo
LCBD
0.1
0.2
0.3
0.4
0.5
NbNp
NbHKSp
NbSp
SbSp
SbHKNp
SbNp
Treatment

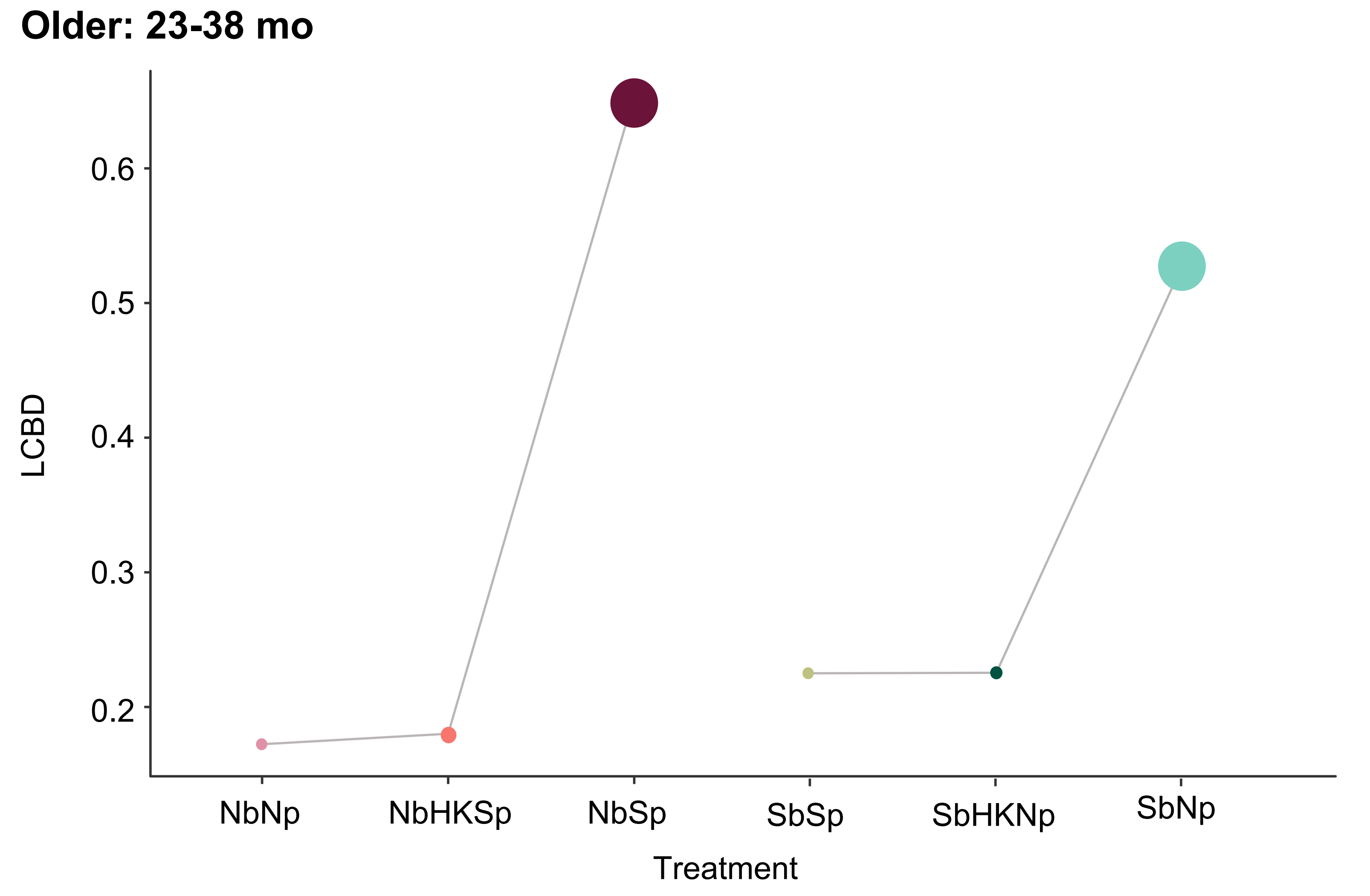
Older: 23-38 mo
LCBD
0.2
0.3
0.4
0.5
0.6
NbNp
NbHKSp
NbSp
SbSp
SbHKNp
SbNp
Treatment

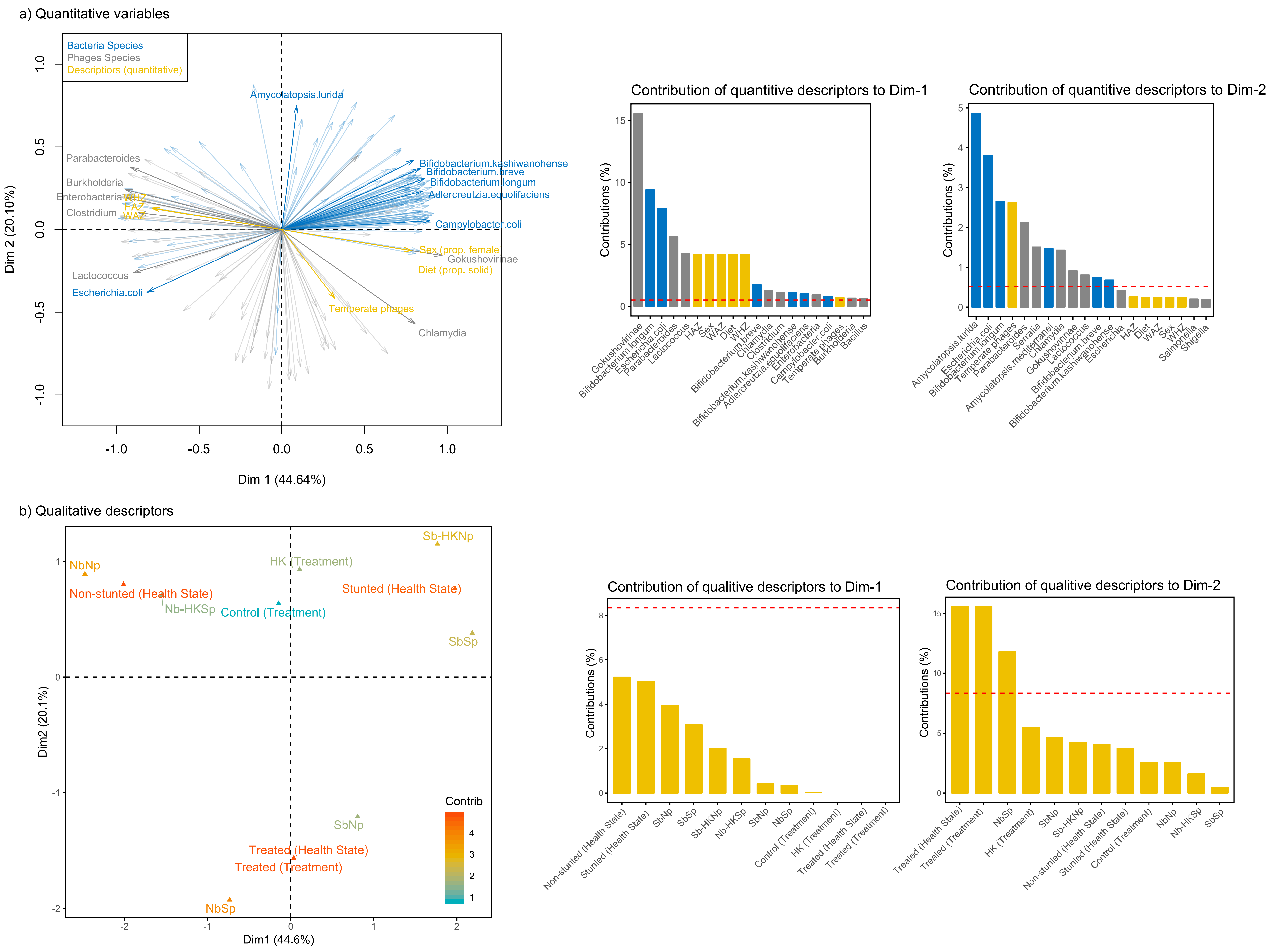
a) Quantitative variables
Bacteria Species
Phages Species
Descriptors (quantitative)
Amycolatopsis.lurida
Parabacteroides
Burkholderia
Enterobacteria
Clostridium
WHZ
HAZ
WAZ
Lactococcus
Escherichia.coli
Bifidobacterium.kashiwanohense
Bifidobacterium.breve
Bifidobacterium.longum
Adlercreutzia.equolifaciens
Campylobacter.coli
Sex (prop. female)
Gokushovirinae
Diet (prop. solid)
Temperate phages
Chlamydia
Dim 1 (44.64%)
Dim 2 (20.10%)
Contribution of quantitive descriptors to Dim-1
Contributions (%)
Gokushovirinae
Bifidobacterium.longum
Escherichia.coli
Parabacteroides
Lactococcus
HAZ
Sex
WAZ
Diet
WHZ
Bifidobacterium.breve
Chlamydia
Clostridium
Bifidobacterium.kashiwanohense
Adlercreutzia.equolifaciens
Enterobacteria
Campylobacter.coli
Temperate phages
Burkholderia
Bacillus
Contribution of quantitive descriptors to Dim-2
Contributions (%)
Amycolatopsis.lurida
Escherichia.coli
Bifidobacterium.longum
Temperate phages
Parabacteroides
Serratia
Amycolatopsis.mediterranei
Chlamydia
Gokushovirinae
Lactococcus
Bifidobacterium.breve
Bifidobacterium.kashiwanohense
Escherichia
HAZ
Diet
WAZ
Sex
WHZ
Salmonella
Shigella
b) Qualitative descriptors
Sb-HKNp
NbNp
HK (Treatment)
Non-stunted (Health State)
Stunted (Health State)
Nb-HKSp
Control (Treatment)
SbSp
SbNp
Treated (Health State)
Treated (Treatment)
NbSp
Contrib
Dim1 (44.6%)
Dim2 (20.1%)
Contribution of qualitive descriptors to Dim-1
Contributions (%)
Non-stunted (Health State)
Stunted (Health State)
SbNp
SbSp
Sb-HKNp
Nb-HKSp
SbNp
NbSp
Control (Treatment)
HK (Treatment)
Treated (Health State)
Treated (Treatment)
Contribution of qualitive descriptors to Dim-2
Contributions (%)
Treated (Health State)
Treated (Treatment)
NbSp
HK (Treatment)
SbNp
Sb-HKNp
Non-stunted (Health State)
Stunted (Health State)
Control (Treatment)
NbNp
Nb-HKSp
SbSp

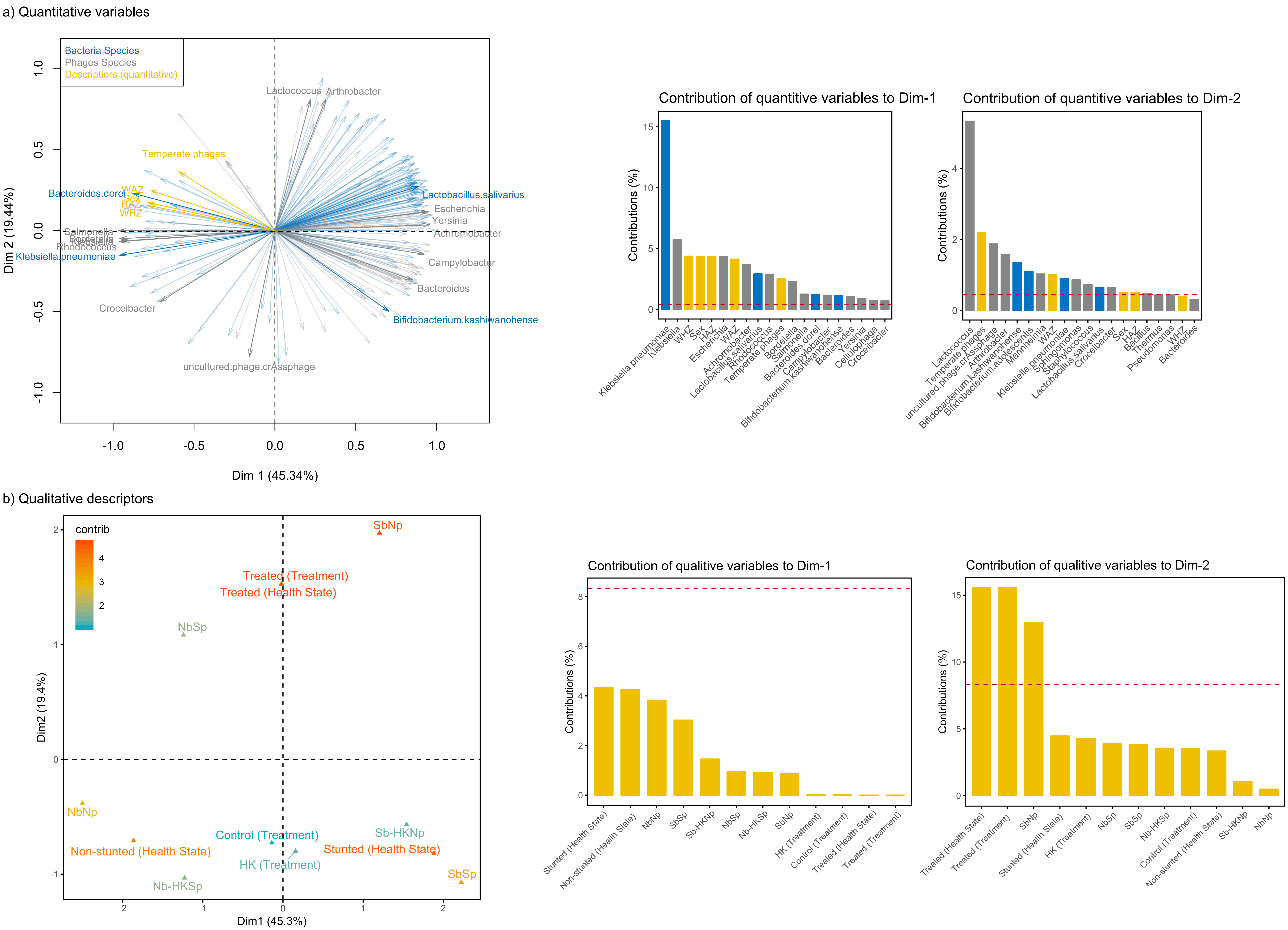

a) Quantitative variables
Bacteria Species
Phages Species
Descriptions (quantitative)
Dim 1 (45.34%)
Dim 2 (19.44%)
Contribution of quantitive variables to Dim-1
Contribution of quantitive variables to Dim-2
Contributions (%)
b) Qualitative descriptors
contrib
Dim1 (45.3%)
Dim2 (19.4%)
Contribution of qualitive variables to Dim-1
Contribution of qualitive variables to Dim-2

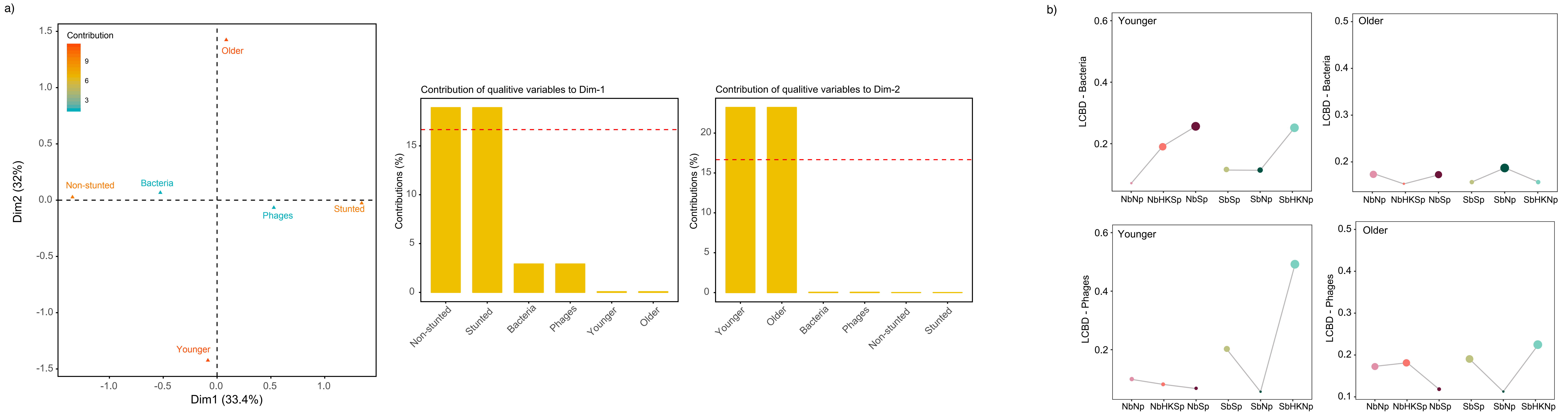

a)
Contribution
9
6
3
Older
Non-stunted
Bacteria
Phages
Stunted
Younger
Dim2 (32%)
Dim1 (33.4%)
Contribution of qualitive variables to Dim-1
Contributions (%)
Non-stunted
Stunted
Bacteria
Phages
Younger
Older
Contribution of qualitive variables to Dim-2
Contributions (%)
Younger
Older
Bacteria
Phages
Non-stunted
Stunted
b)
Younger
Older
LCBD - Bacteria
LCBD - Phages
NbNp
NbHKSp
NbSp
SbSp
SbNp
SbHKNp
